# Distributions of topological observables in inclusive three- and four-jet events in pp collisions at $$\sqrt{s} = 7$$$$\,\text {TeV}$$

**DOI:** 10.1140/epjc/s10052-015-3491-9

**Published:** 2015-07-01

**Authors:** V. Khachatryan, A. M. Sirunyan, A. Tumasyan, W. Adam, T. Bergauer, M. Dragicevic, J. Erö, M. Friedl, R. Frühwirth, V. M. Ghete, C. Hartl, N. Hörmann, J. Hrubec, M. Jeitler, W. Kiesenhofer, V. Knünz, M. Krammer, I. Krätschmer, D. Liko, I. Mikulec, D. Rabady, B. Rahbaran, H. Rohringer, R. Schöfbeck, J. Strauss, W. Treberer-Treberspurg, W. Waltenberger, C.-E. Wulz, V. Mossolov, N. Shumeiko, J. Suarez Gonzalez, S. Alderweireldt, S. Bansal, T. Cornelis, E. A. De Wolf, X. Janssen, A. Knutsson, J. Lauwers, S. Luyckx, S. Ochesanu, R. Rougny, M. Van De Klundert, H. Van Haevermaet, P. Van Mechelen, N. Van Remortel, A. Van Spilbeeck, F. Blekman, S. Blyweert, J. D’Hondt, N. Daci, N. Heracleous, J. Keaveney, S. Lowette, M. Maes, A. Olbrechts, Q. Python, D. Strom, S. Tavernier, W. Van Doninck, P. Van Mulders, G. P. Van Onsem, I. Villella, C. Caillol, B. Clerbaux, G. De Lentdecker, D. Dobur, L. Favart, A. P. R. Gay, A. Grebenyuk, A. Léonard, A. Mohammadi, L. Perniè, A. Randle-conde, T. Reis, T. Seva, L. Thomas, C. Vander Velde, P. Vanlaer, J. Wang, F. Zenoni, V. Adler, K. Beernaert, L. Benucci, A. Cimmino, S. Costantini, S. Crucy, S. Dildick, A. Fagot, G. Garcia, J. Mccartin, A. A. Ocampo Rios, D. Ryckbosch, S. Salva Diblen, M. Sigamani, N. Strobbe, F. Thyssen, M. Tytgat, E. Yazgan, N. Zaganidis, S. Basegmez, C. Beluffi, G. Bruno, R. Castello, A. Caudron, L. Ceard, G. G. Da Silveira, C. Delaere, T. du Pree, D. Favart, L. Forthomme, A. Giammanco, J. Hollar, A. Jafari, P. Jez, M. Komm, V. Lemaitre, C. Nuttens, D. Pagano, L. Perrini, A. Pin, K. Piotrzkowski, A. Popov, L. Quertenmont, M. Selvaggi, M. Vidal Marono, J. M. Vizan Garcia, N. Beliy, T. Caebergs, E. Daubie, G. H. Hammad, W. L. Aldá Júnior, G. A. Alves, L. Brito, M. Correa Martins Junior, T. Dos Reis Martins, C. Mora Herrera, M. E. Pol, P. Rebello Teles, W. Carvalho, J. Chinellato, A. Custódio, E. M. Da Costa, D. De Jesus Damiao, C. De Oliveira Martins, S. Fonseca De Souza, H. Malbouisson, D. Matos Figueiredo, L. Mundim, H. Nogima, W. L. Prado Da Silva, J. Santaolalla, A. Santoro, A. Sznajder, E. J. Tonelli Manganote, A. Vilela Pereira, C. A. Bernardes, S. Dogra, T. R. Fernandez Perez Tomei, E. M. Gregores, P. G. Mercadante, S. F. Novaes, Sandra S. Padula, A. Aleksandrov, V. Genchev, R. Hadjiiska, P. Iaydjiev, A. Marinov, S. Piperov, M. Rodozov, G. Sultanov, M. Vutova, A. Dimitrov, I. Glushkov, L. Litov, B. Pavlov, P. Petkov, J. G. Bian, G. M. Chen, H. S. Chen, M. Chen, T. Cheng, R. Du, C. H. Jiang, R. Plestina, F. Romeo, J. Tao, Z. Wang, C. Asawatangtrakuldee, Y. Ban, Q. Li, S. Liu, Y. Mao, S. J. Qian, D. Wang, Z. Xu, W. Zou, C. Avila, A. Cabrera, L. F. Chaparro Sierra, C. Florez, J. P. Gomez, B. Gomez Moreno, J. C. Sanabria, N. Godinovic, D. Lelas, D. Polic, I. Puljak, Z. Antunovic, M. Kovac, V. Brigljevic, K. Kadija, J. Luetic, D. Mekterovic, L. Sudic, A. Attikis, G. Mavromanolakis, J. Mousa, C. Nicolaou, F. Ptochos, P. A. Razis, M. Bodlak, M. Finger, M. Finger, Y. Assran, A. Ellithi Kamel, M. A. Mahmoud, A. Radi, M. Kadastik, M. Murumaa, M. Raidal, A. Tiko, P. Eerola, G. Fedi, M. Voutilainen, J. Härkönen, V. Karimäki, R. Kinnunen, M. J. Kortelainen, T. Lampén, K. Lassila-Perini, S. Lehti, T. Lindén, P. Luukka, T. Mäenpää, T. Peltola, E. Tuominen, J. Tuominiemi, E. Tuovinen, L. Wendland, J. Talvitie, T. Tuuva, M. Besancon, F. Couderc, M. Dejardin, D. Denegri, B. Fabbro, J. L. Faure, C. Favaro, F. Ferri, S. Ganjour, A. Givernaud, P. Gras, G. Hamel de Monchenault, P. Jarry, E. Locci, J. Malcles, J. Rander, A. Rosowsky, M. Titov, S. Baffioni, F. Beaudette, P. Busson, C. Charlot, T. Dahms, M. Dalchenko, L. Dobrzynski, N. Filipovic, A. Florent, R. Granier de Cassagnac, L. Mastrolorenzo, P. Miné, C. Mironov, I. N. Naranjo, M. Nguyen, C. Ochando, G. Ortona, P. Paganini, S. Regnard, R. Salerno, J. B. Sauvan, Y. Sirois, C. Veelken, Y. Yilmaz, A. Zabi, J.-L. Agram, J. Andrea, A. Aubin, D. Bloch, J.-M. Brom, E. C. Chabert, C. Collard, E. Conte, J.-C. Fontaine, D. Gelé, U. Goerlach, C. Goetzmann, A.-C. Le Bihan, K. Skovpen, P. Van Hove, S. Gadrat, S. Beauceron, N. Beaupere, G. Boudoul, E. Bouvier, S. Brochet, C. A. Carrillo Montoya, J. Chasserat, R. Chierici, D. Contardo, P. Depasse, H. El Mamouni, J. Fan, J. Fay, S. Gascon, M. Gouzevitch, B. Ille, T. Kurca, M. Lethuillier, L. Mirabito, S. Perries, J. D. Ruiz Alvarez, D. Sabes, L. Sgandurra, V. Sordini, M. Vander Donckt, P. Verdier, S. Viret, H. Xiao, Z. Tsamalaidze, C. Autermann, S. Beranek, M. Bontenackels, M. Edelhoff, L. Feld, A. Heister, O. Hindrichs, K. Klein, A. Ostapchuk, F. Raupach, J. Sammet, S. Schael, J. F. Schulte, H. Weber, B. Wittmer, V. Zhukov, M. Ata, M. Brodski, E. Dietz-Laursonn, D. Duchardt, M. Erdmann, R. Fischer, A. Güth, T. Hebbeker, C. Heidemann, K. Hoepfner, D. Klingebiel, S. Knutzen, P. Kreuzer, M. Merschmeyer, A. Meyer, P. Millet, M. Olschewski, K. Padeken, P. Papacz, H. Reithler, S. A. Schmitz, L. Sonnenschein, D. Teyssier, S. Thüer, M. Weber, V. Cherepanov, Y. Erdogan, G. Flügge, H. Geenen, M. Geisler, W. Haj Ahmad, F. Hoehle, B. Kargoll, T. Kress, Y. Kuessel, A. Künsken, J. Lingemann, A. Nowack, I. M. Nugent, O. Pooth, A. Stahl, M. Aldaya Martin, I. Asin, N. Bartosik, J. Behr, U. Behrens, A. J. Bell, A. Bethani, K. Borras, A. Burgmeier, A. Cakir, L. Calligaris, A. Campbell, S. Choudhury, F. Costanza, C. Diez Pardos, G. Dolinska, S. Dooling, T. Dorland, G. Eckerlin, D. Eckstein, T. Eichhorn, G. Flucke, J. Garay Garcia, A. Geiser, P. Gunnellini, J. Hauk, M. Hempel, H. Jung, A. Kalogeropoulos, M. Kasemann, P. Katsas, J. Kieseler, C. Kleinwort, l. Korol, D. Krücker, W. Lange, J. Leonard, K. Lipka, A. Lobanov, W. Lohmann, B. Lutz, R. Mankel, I. Marfin, I.-A. Melzer-Pellmann, A. B. Meyer, G. Mittag, J. Mnich, A. Mussgiller, S. Naumann-Emme, A. Nayak, E. Ntomari, H. Perrey, D. Pitzl, R. Placakyte, A. Raspereza, P. M. Ribeiro Cipriano, B. Roland, E. Ron, M. Ö. Sahin, J. Salfeld-Nebgen, P. Saxena, T. Schoerner-Sadenius, M. Schröder, C. Seitz, S. Spannagel, A. D. R. Vargas Trevino, R. Walsh, C. Wissing, V. Blobel, M. Centis Vignali, A. R. Draeger, J. Erfle, E. Garutti, K. Goebel, M. Görner, J. Haller, M. Hoffmann, R. S. Höing, A. Junkes, H. Kirschenmann, R. Klanner, R. Kogler, J. Lange, T. Lapsien, T. Lenz, I. Marchesini, J. Ott, T. Peiffer, A. Perieanu, N. Pietsch, J. Poehlsen, T. Poehlsen, D. Rathjens, C. Sander, H. Schettler, P. Schleper, E. Schlieckau, A. Schmidt, M. Seidel, V. Sola, H. Stadie, G. Steinbrück, D. Troendle, E. Usai, L. Vanelderen, A. Vanhoefer, C. Barth, C. Baus, J. Berger, C. Böser, E. Butz, T. Chwalek, W. De Boer, A. Descroix, A. Dierlamm, M. Feindt, F. Frensch, M. Giffels, A. Gilbert, F. Hartmann, T. Hauth, U. Husemann, I. Katkov, A. Kornmayer, E. Kuznetsova, P. Lobelle Pardo, M. U. Mozer, T. Müller, Th. Müller, A. Nürnberg, G. Quast, K. Rabbertz, S. Röcker, H. J. Simonis, F. M. Stober, R. Ulrich, J. Wagner-Kuhr, S. Wayand, T. Weiler, R. Wolf, G. Anagnostou, G. Daskalakis, T. Geralis, V. A. Giakoumopoulou, A. Kyriakis, D. Loukas, A. Markou, C. Markou, A. Psallidas, I. Topsis-Giotis, A. Agapitos, S. Kesisoglou, A. Panagiotou, N. Saoulidou, E. Stiliaris, X. Aslanoglou, I. Evangelou, G. Flouris, C. Foudas, P. Kokkas, N. Manthos, I. Papadopoulos, E. Paradas, J. Strologas, G. Bencze, C. Hajdu, P. Hidas, D. Horvath, F. Sikler, V. Veszpremi, G. Vesztergombi, A. J. Zsigmond, N. Beni, S. Czellar, J. Karancsi, J. Molnar, J. Palinkas, Z. Szillasi, A. Makovec, P. Raics, Z. L. Trocsanyi, B. Ujvari, S. K. Swain, S. B. Beri, V. Bhatnagar, R. Gupta, U. Bhawandeep, A. K. Kalsi, M. Kaur, R. Kumar, M. Mittal, N. Nishu, J. B. Singh, Ashok Kumar, Arun Kumar, S. Ahuja, A. Bhardwaj, B. C. Choudhary, A. Kumar, S. Malhotra, M. Naimuddin, K. Ranjan, V. Sharma, S. Banerjee, S. Bhattacharya, K. Chatterjee, S. Dutta, B. Gomber, Sa. Jain, Sh. Jain, R. Khurana, A. Modak, S. Mukherjee, D. Roy, S. Sarkar, M. Sharan, A. Abdulsalam, D. Dutta, V. Kumar, A. K. Mohanty, L. M. Pant, P. Shukla, A. Topkar, T. Aziz, S. Banerjee, S. Bhowmik, R. M. Chatterjee, R. K. Dewanjee, S. Dugad, S. Ganguly, S. Ghosh, M. Guchait, A. Gurtu, G. Kole, S. Kumar, M. Maity, G. Majumder, K. Mazumdar, G. B. Mohanty, B. Parida, K. Sudhakar, N. Wickramage, H. Bakhshiansohi, H. Behnamian, S. M. Etesami, A. Fahim, R. Goldouzian, M. Khakzad, M. Mohammadi Najafabadi, M. Naseri, S. Paktinat Mehdiabadi, F. Rezaei Hosseinabadi, B. Safarzadeh, M. Zeinali, M. Felcini, M. Grunewald, M. Abbrescia, C. Calabria, S. S. Chhibra, A. Colaleo, D. Creanza, N. De Filippis, M. De Palma, L. Fiore, G. Iaselli, G. Maggi, M. Maggi, S. My, S. Nuzzo, A. Pompili, G. Pugliese, R. Radogna, G. Selvaggi, A. Sharma, L. Silvestris, R. Venditti, P. Verwilligen, G. Abbiendi, A. C. Benvenuti, D. Bonacorsi, S. Braibant-Giacomelli, L. Brigliadori, R. Campanini, P. Capiluppi, A. Castro, F. R. Cavallo, G. Codispoti, M. Cuffiani, G. M. Dallavalle, F. Fabbri, A. Fanfani, D. Fasanella, P. Giacomelli, C. Grandi, L. Guiducci, S. Marcellini, G. Masetti, A. Montanari, F. L. Navarria, A. Perrotta, A. M. Rossi, F. Primavera, T. Rovelli, G. P. Siroli, N. Tosi, R. Travaglini, S. Albergo, G. Cappello, M. Chiorboli, S. Costa, F. Giordano, R. Potenza, A. Tricomi, C. Tuve, G. Barbagli, V. Ciulli, C. Civinini, R. D’Alessandro, E. Focardi, E. Gallo, S. Gonzi, V. Gori, P. Lenzi, M. Meschini, S. Paoletti, G. Sguazzoni, A. Tropiano, L. Benussi, S. Bianco, F. Fabbri, D. Piccolo, R. Ferretti, F. Ferro, M. Lo Vetere, E. Robutti, S. Tosi, M. E. Dinardo, S. Fiorendi, S. Gennai, R. Gerosa, A. Ghezzi, P. Govoni, M. T. Lucchini, S. Malvezzi, R. A. Manzoni, A. Martelli, B. Marzocchi, D. Menasce, L. Moroni, M. Paganoni, D. Pedrini, S. Ragazzi, N. Redaelli, T. Tabarelli de Fatis, S. Buontempo, N. Cavallo, S. Di Guida, F. Fabozzi, A. O. M. Iorio, L. Lista, S. Meola, M. Merola, P. Paolucci, P. Azzi, N. Bacchetta, M. Bellato, M. Biasotto, M. Dall’Osso, T. Dorigo, M. Galanti, P. Giubilato, F. Gonella, A. Gozzelino, K. Kanishchev, S. Lacaprara, M. Margoni, A. T. Meneguzzo, F. Montecassiano, M. Passaseo, J. Pazzini, M. Pegoraro, N. Pozzobon, P. Ronchese, F. Simonetto, E. Torassa, M. Tosi, S. Vanini, S. Ventura, P. Zotto, A. Zucchetta, M. Gabusi, S. P. Ratti, V. Re, C. Riccardi, P. Salvini, P. Vitulo, M. Biasini, G. M. Bilei, D. Ciangottini, L. Fanò, P. Lariccia, G. Mantovani, M. Menichelli, A. Saha, A. Santocchia, A. Spiezia, K. Androsov, P. Azzurri, G. Bagliesi, J. Bernardini, T. Boccali, G. Broccolo, R. Castaldi, M. A. Ciocci, R. Dell’Orso, S. Donato, G. Fedi, F. Fiori, L. Foà, A. Giassi, M. T. Grippo, F. Ligabue, T. Lomtadze, L. Martini, A. Messineo, C. S. Moon, F. Palla, A. Rizzi, A. Savoy-Navarro, A. T. Serban, P. Spagnolo, P. Squillacioti, R. Tenchini, G. Tonelli, A. Venturi, P. G. Verdini, C. Vernieri, L. Barone, F. Cavallari, G. D’imperio, D. Del Re, M. Diemoz, C. Jorda, E. Longo, F. Margaroli, P. Meridiani, F. Micheli, S. Nourbakhsh, G. Organtini, R. Paramatti, S. Rahatlou, C. Rovelli, F. Santanastasio, L. Soffi, P. Traczyk, N. Amapane, R. Arcidiacono, S. Argiro, M. Arneodo, R. Bellan, C. Biino, N. Cartiglia, S. Casasso, M. Costa, A. Degano, N. Demaria, L. Finco, C. Mariotti, S. Maselli, E. Migliore, V. Monaco, M. Musich, M. M. Obertino, L. Pacher, N. Pastrone, M. Pelliccioni, G. L. Pinna Angioni, A. Potenza, A. Romero, M. Ruspa, R. Sacchi, A. Solano, A. Staiano, U. Tamponi, S. Belforte, V. Candelise, M. Casarsa, F. Cossutti, G. Della Ricca, B. Gobbo, C. La Licata, M. Marone, A. Schizzi, T. Umer, A. Zanetti, S. Chang, T. A. Kropivnitskaya, S. K. Nam, D. H. Kim, G. N. Kim, M. S. Kim, M. S. Kim, D. J. Kong, S. Lee, Y. D. Oh, H. Park, A. Sakharov, D. C. Son, T. J. Kim, J. Y. Kim, S. Song, S. Choi, D. Gyun, B. Hong, M. Jo, H. Kim, Y. Kim, B. Lee, K. S. Lee, S. K. Park, Y. Roh, H. D. Yoo, M. Choi, J. H. Kim, I. C. Park, G. Ryu, M. S. Ryu, Y. Choi, Y. K. Choi, J. Goh, D. Kim, E. Kwon, J. Lee, I. Yu, A. Juodagalvis, J. R. Komaragiri, M. A. B. Md Ali, E. Casimiro Linares, H. Castilla-Valdez, E. De La Cruz-Burelo, I. Heredia-de La Cruz, A. Hernandez-Almada, R. Lopez-Fernandez, A. Sanchez-Hernandez, S. Carrillo Moreno, F. Vazquez Valencia, I. Pedraza, H. A. Salazar Ibarguen, A. Morelos Pineda, D. Krofcheck, P. H. Butler, S. Reucroft, A. Ahmad, M. Ahmad, Q. Hassan, H. R. Hoorani, W. A. Khan, T. Khurshid, M. Shoaib, H. Bialkowska, M. Bluj, B. Boimska, T. Frueboes, M. Górski, M. Kazana, K. Nawrocki, K. Romanowska-Rybinska, M. Szleper, P. Zalewski, G. Brona, K. Bunkowski, M. Cwiok, W. Dominik, K. Doroba, A. Kalinowski, M. Konecki, J. Krolikowski, M. Misiura, M. Olszewski, W. Wolszczak, P. Bargassa, C. Beir ao Da Cruz E Silva, P. Faccioli, P. G. Ferreira Parracho, M. Gallinaro, L. Lloret Iglesias, F. Nguyen, J. Rodrigues Antunes, J. Seixas, J. Varela, P. Vischia, S. Afanasiev, P. Bunin, M. Gavrilenko, I. Golutvin, I. Gorbunov, A. Kamenev, V. Karjavin, V. Konoplyanikov, A. Lanev, A. Malakhov, V. Matveev, P. Moisenz, V. Palichik, V. Perelygin, S. Shmatov, N. Skatchkov, V. Smirnov, A. Zarubin, V. Golovtsov, Y. Ivanov, V. Kim, P. Levchenko, V. Murzin, V. Oreshkin, I. Smirnov, V. Sulimov, L. Uvarov, S. Vavilov, A. Vorobyev, An. Vorobyev, Yu. Andreev, A. Dermenev, S. Gninenko, N. Golubev, M. Kirsanov, N. Krasnikov, A. Pashenkov, D. Tlisov, A. Toropin, V. Epshteyn, V. Gavrilov, N. Lychkovskaya, V. Popov, l. Pozdnyakov, G. Safronov, S. Semenov, A. Spiridonov, V. Stolin, E. Vlasov, A. Zhokin, V. Andreev, M. Azarkin, I. Dremin, M. Kirakosyan, A. Leonidov, G. Mesyats, S. V. Rusakov, A. Vinogradov, A. Belyaev, E. Boos, M. Dubinin, L. Dudko, A. Ershov, A. Gribushin, V. Klyukhin, O. Kodolova, I. Lokhtin, S. Obraztsov, S. Petrushanko, V. Savrin, A. Snigirev, I. Azhgirey, I. Bayshev, S. Bitioukov, V. Kachanov, A. Kalinin, D. Konstantinov, V. Krychkine, V. Petrov, R. Ryutin, A. Sobol, L. Tourtchanovitch, S. Troshin, N. Tyurin, A. Uzunian, A. Volkov, P. Adzic, M. Ekmedzic, J. Milosevic, V. Rekovic, J. Alcaraz Maestre, C. Battilana, E. Calvo, M. Cerrada, M. Chamizo Llatas, N. Colino, B. De La Cruz, A. Delgado Peris, D. Domínguez Vázquez, A. Escalante Del Valle, C. Fernandez Bedoya, J. P. Fernández Ramos, J. Flix, M. C. Fouz, P. Garcia-Abia, O. Gonzalez Lopez, S. Goy Lopez, J. M. Hernandez, M. I. Josa, E. Navarro De Martino, A. Pérez-Calero Yzquierdo, J. Puerta Pelayo, A. Quintario Olmeda, I. Redondo, L. Romero, M. S. Soares, C. Albajar, J. F. de Trocóniz, M. Missiroli, D. Moran, H. Brun, J. Cuevas, J. Fernandez Menendez, S. Folgueras, I. Gonzalez Caballero, J. A. Brochero Cifuentes, I. J. Cabrillo, A. Calderon, J. Duarte Campderros, M. Fernandez, G. Gomez, A. Graziano, A. Lopez Virto, J. Marco, R. Marco, C. Martinez Rivero, F. Matorras, F. J. Munoz Sanchez, J. Piedra Gomez, T. Rodrigo, A. Y. Rodríguez-Marrero, A. Ruiz-Jimeno, L. Scodellaro, I. Vila, R. Vilar Cortabitarte, D. Abbaneo, E. Auffray, G. Auzinger, M. Bachtis, P. Baillon, A. H. Ball, D. Barney, A. Benaglia, J. Bendavid, L. Benhabib, J. F. Benitez, C. Bernet, P. Bloch, A. Bocci, A. Bonato, O. Bondu, C. Botta, H. Breuker, T. Camporesi, G. Cerminara, S. Colafranceschi, M. D’Alfonso, D. d’Enterria, A. Dabrowski, A. David, F. De Guio, A. De Roeck, S. De Visscher, E. Di Marco, M. Dobson, M. Dordevic, B. Dorney, N. Dupont-Sagorin, A. Elliott-Peisert, G. Franzoni, W. Funk, D. Gigi, K. Gill, D. Giordano, M. Girone, F. Glege, R. Guida, S. Gundacker, M. Guthoff, J. Hammer, M. Hansen, P. Harris, J. Hegeman, V. Innocente, P. Janot, K. Kousouris, K. Krajczar, P. Lecoq, C. Lourenço, N. Magini, L. Malgeri, M. Mannelli, J. Marrouche, L. Masetti, F. Meijers, S. Mersi, E. Meschi, F. Moortgat, S. Morovic, M. Mulders, L. Orsini, L. Pape, E. Perez, L. Perrozzi, A. Petrilli, G. Petrucciani, A. Pfeiffer, M. Pimiä, D. Piparo, M. Plagge, A. Racz, G. Rolandi, M. Rovere, H. Sakulin, C. Schäfer, C. Schwick, A. Sharma, P. Siegrist, P. Silva, M. Simon, P. Sphicas, D. Spiga, J. Steggemann, B. Stieger, M. Stoye, Y. Takahashi, D. Treille, A. Tsirou, G. I. Veres, N. Wardle, H. K. Wöhri, H. Wollny, W. D. Zeuner, W. Bertl, K. Deiters, W. Erdmann, R. Horisberger, Q. Ingram, H. C. Kaestli, D. Kotlinski, U. Langenegger, D. Renker, T. Rohe, F. Bachmair, L. Bäni, L. Bianchini, M. A. Buchmann, B. Casal, N. Chanon, G. Dissertori, M. Dittmar, M. Donegà, M. Dünser, P. Eller, C. Grab, D. Hits, J. Hoss, W. Lustermann, B. Mangano, A. C. Marini, M. Marionneau, P. Martinez Ruiz del Arbol, M. Masciovecchio, D. Meister, N. Mohr, P. Musella, C. Nägeli, F. Nessi-Tedaldi, F. Pandolfi, F. Pauss, M. Peruzzi, M. Quittnat, L. Rebane, M. Rossini, A. Starodumov, M. Takahashi, K. Theofilatos, R. Wallny, H. A. Weber, C. Amsler, M. F. Canelli, V. Chiochia, A. De Cosa, A. Hinzmann, T. Hreus, B. Kilminster, C. Lange, B. Millan Mejias, J. Ngadiuba, D. Pinna, P. Robmann, F. J. Ronga, S. Taroni, M. Verzetti, Y. Yang, M. Cardaci, K. H. Chen, C. Ferro, C. M. Kuo, W. Lin, Y. J. Lu, R. Volpe, S. S. Yu, P. Chang, Y. H. Chang, Y. W. Chang, Y. Chao, K. F. Chen, P. H. Chen, C. Dietz, U. Grundler, W.-S. Hou, K. Y. Kao, Y. F. Liu, R.-S. Lu, D. Majumder, E. Petrakou, Y. M. Tzeng, R. Wilken, B. Asavapibhop, G. Singh, N. Srimanobhas, N. Suwonjandee, A. Adiguzel, M. N. Bakirci, S. Cerci, C. Dozen, I. Dumanoglu, E. Eskut, S. Girgis, G. Gokbulut, E. Gurpinar, I. Hos, E. E. Kangal, A. Kayis Topaksu, G. Onengut, K. Ozdemir, S. Ozturk, A. Polatoz, D. Sunar Cerci, B. Tali, H. Topakli, M. Vergili, I. V. Akin, B. Bilin, S. Bilmis, H. Gamsizkan, B. Isildak, G. Karapinar, K. Ocalan, S. Sekmen, U. E. Surat, M. Yalvac, M. Zeyrek, A. Albayrak, E. Gülmez, M. Kaya, O. Kaya, T. Yetkin, K. Cankocak, F. I. Vardarlı, L. Levchuk, P. Sorokin, J. J. Brooke, E. Clement, D. Cussans, H. Flacher, J. Goldstein, M. Grimes, G. P. Heath, H. F. Heath, J. Jacob, L. Kreczko, C. Lucas, Z. Meng, D. M. Newbold, S. Paramesvaran, A. Poll, T. Sakuma, S. Senkin, V. J. Smith, K. W. Bell, A. Belyaev, C. Brew, R. M. Brown, D. J. A. Cockerill, J. A. Coughlan, K. Harder, S. Harper, E. Olaiya, D. Petyt, C. H. Shepherd-Themistocleous, A. Thea, I. R. Tomalin, T. Williams, W. J. Womersley, S. D. Worm, M. Baber, R. Bainbridge, O. Buchmuller, D. Burton, D. Colling, N. Cripps, P. Dauncey, G. Davies, M. Della Negra, P. Dunne, W. Ferguson, J. Fulcher, D. Futyan, G. Hall, G. Iles, M. Jarvis, G. Karapostoli, M. Kenzie, R. Lane, R. Lucas, L. Lyons, A.-M. Magnan, S. Malik, B. Mathias, J. Nash, A. Nikitenko, J. Pela, M. Pesaresi, K. Petridis, D. M. Raymond, S. Rogerson, A. Rose, C. Seez, P. Sharp, A. Tapper, M. Vazquez Acosta, T. Virdee, S. C. Zenz, J. E. Cole, P. R. Hobson, A. Khan, P. Kyberd, D. Leggat, D. Leslie, I. D. Reid, P. Symonds, L. Teodorescu, M. Turner, J. Dittmann, K. Hatakeyama, A. Kasmi, H. Liu, T. Scarborough, O. Charaf, S. I. Cooper, C. Henderson, P. Rumerio, A. Avetisyan, T. Bose, C. Fantasia, P. Lawson, C. Richardson, J. Rohlf, J. St. John, L. Sulak, J. Alimena, E. Berry, S. Bhattacharya, G. Christopher, D. Cutts, Z. Demiragli, N. Dhingra, A. Ferapontov, A. Garabedian, U. Heintz, G. Kukartsev, E. Laird, G. Landsberg, M. Luk, M. Narain, M. Segala, T. Sinthuprasith, T. Speer, J. Swanson, R. Breedon, G. Breto, M. Calderon De La Barca Sanchez, S. Chauhan, M. Chertok, J. Conway, R. Conway, P. T. Cox, R. Erbacher, M. Gardner, W. Ko, R. Lander, M. Mulhearn, D. Pellett, J. Pilot, F. Ricci-Tam, S. Shalhout, J. Smith, M. Squires, D. Stolp, M. Tripathi, S. Wilbur, R. Yohay, R. Cousins, P. Everaerts, C. Farrell, J. Hauser, M. Ignatenko, G. Rakness, E. Takasugi, V. Valuev, M. Weber, K. Burt, R. Clare, J. Ellison, J. W. Gary, G. Hanson, J. Heilman, M. Ivova Rikova, P. Jandir, E. Kennedy, F. Lacroix, O. R. Long, A. Luthra, M. Malberti, M. Olmedo Negrete, A. Shrinivas, S. Sumowidagdo, S. Wimpenny, J. G. Branson, G. B. Cerati, S. Cittolin, R. T. D’Agnolo, A. Holzner, R. Kelley, D. Klein, J. Letts, I. Macneill, D. Olivito, S. Padhi, C. Palmer, M. Pieri, M. Sani, V. Sharma, S. Simon, M. Tadel, Y. Tu, A. Vartak, C. Welke, F. Würthwein, A. Yagil, D. Barge, J. Bradmiller-Feld, C. Campagnari, T. Danielson, A. Dishaw, V. Dutta, K. Flowers, M. Franco Sevilla, P. Geffert, C. George, F. Golf, L. Gouskos, J. Incandela, C. Justus, N. Mccoll, J. Richman, D. Stuart, W. To, C. West, J. Yoo, A. Apresyan, A. Bornheim, J. Bunn, Y. Chen, J. Duarte, A. Mott, H. B. Newman, C. Pena, M. Pierini, M. Spiropulu, J. R. Vlimant, R. Wilkinson, S. Xie, R. Y. Zhu, V. Azzolini, A. Calamba, B. Carlson, T. Ferguson, Y. Iiyama, M. Paulini, J. Russ, H. Vogel, I. Vorobiev, J. P. Cumalat, W. T. Ford, A. Gaz, M. Krohn, E. Luiggi Lopez, U. Nauenberg, J. G. Smith, K. Stenson, S. R. Wagner, J. Alexander, A. Chatterjee, J. Chaves, J. Chu, S. Dittmer, N. Eggert, N. Mirman, G. Nicolas Kaufman, J. R. Patterson, A. Ryd, E. Salvati, L. Skinnari, W. Sun, W. D. Teo, J. Thom, J. Thompson, J. Tucker, Y. Weng, L. Winstrom, P. Wittich, D. Winn, S. Abdullin, M. Albrow, J. Anderson, G. Apollinari, L. A. T. Bauerdick, A. Beretvas, J. Berryhill, P. C. Bhat, G. Bolla, K. Burkett, J. N. Butler, H. W. K. Cheung, F. Chlebana, S. Cihangir, V. D. Elvira, I. Fisk, J. Freeman, Y. Gao, E. Gottschalk, L. Gray, D. Green, S. Grünendahl, O. Gutsche, J. Hanlon, D. Hare, R. M. Harris, J. Hirschauer, B. Hooberman, S. Jindariani, M. Johnson, U. Joshi, K. Kaadze, B. Klima, B. Kreis, S. Kwan, J. Linacre, D. Lincoln, R. Lipton, T. Liu, J. Lykken, K. Maeshima, J. M. Marraffino, V. I. Martinez Outschoorn, S. Maruyama, D. Mason, P. McBride, P. Merkel, K. Mishra, S. Mrenna, S. Nahn, C. Newman-Holmes, V. O’Dell, O. Prokofyev, E. Sexton-Kennedy, S. Sharma, A. Soha, W. J. Spalding, L. Spiegel, L. Taylor, S. Tkaczyk, N. V. Tran, L. Uplegger, E. W. Vaandering, R. Vidal, A. Whitbeck, J. Whitmore, F. Yang, D. Acosta, P. Avery, P. Bortignon, D. Bourilkov, M. Carver, D. Curry, S. Das, M. De Gruttola, G. P. Di Giovanni, R. D. Field, M. Fisher, I. K. Furic, J. Hugon, J. Konigsberg, A. Korytov, T. Kypreos, J. F. Low, K. Matchev, H. Mei, P. Milenovic, G. Mitselmakher, L. Muniz, A. Rinkevicius, L. Shchutska, M. Snowball, D. Sperka, J. Yelton, M. Zakaria, S. Hewamanage, S. Linn, P. Markowitz, G. Martinez, J. L. Rodriguez, T. Adams, A. Askew, J. Bochenek, B. Diamond, J. Haas, S. Hagopian, V. Hagopian, K. F. Johnson, H. Prosper, V. Veeraraghavan, M. Weinberg, M. M. Baarmand, M. Hohlmann, H. Kalakhety, F. Yumiceva, M. R. Adams, L. Apanasevich, D. Berry, R. R. Betts, I. Bucinskaite, R. Cavanaugh, O. Evdokimov, L. Gauthier, C. E. Gerber, D. J. Hofman, P. Kurt, D. H. Moon, C. O’Brien, l. D. Sandoval Gonzalez, C. Silkworth, P. Turner, N. Varelas, B. Bilki, W. Clarida, K. Dilsiz, M. Haytmyradov, J.-P. Merlo, H. Mermerkaya, A. Mestvirishvili, A. Moeller, J. Nachtman, H. Ogul, Y. Onel, F. Ozok, A. Penzo, R. Rahmat, S. Sen, P. Tan, E. Tiras, J. Wetzel, K. Yi, B. A. Barnett, B. Blumenfeld, S. Bolognesi, D. Fehling, A. V. Gritsan, P. Maksimovic, C. Martin, M. Swartz, P. Baringer, A. Bean, G. Benelli, C. Bruner, R. P. Kenny, M. Malek, M. Murray, D. Noonan, S. Sanders, J. Sekaric, R. Stringer, Q. Wang, J. S. Wood, I. Chakaberia, A. Ivanov, S. Khalil, M. Makouski, Y. Maravin, L. K. Saini, N. Skhirtladze, I. Svintradze, J. Gronberg, D. Lange, F. Rebassoo, D. Wright, A. Baden, A. Belloni, B. Calvert, S. C. Eno, J. A. Gomez, N. J. Hadley, R. G. Kellogg, T. Kolberg, Y. Lu, A. C. Mignerey, K. Pedro, A. Skuja, M. B. Tonjes, S. C. Tonwar, A. Apyan, R. Barbieri, W. Busza, I. A. Cali, M. Chan, L. Di Matteo, G. Gomez Ceballos, M. Goncharov, D. Gulhan, M. Klute, Y. S. Lai, Y.-J. Lee, A. Levin, P. D. Luckey, C. Paus, D. Ralph, C. Roland, G. Roland, G. S. F. Stephans, K. Sumorok, D. Velicanu, J. Veverka, B. Wyslouch, M. Yang, M. Zanetti, V. Zhukova, B. Dahmes, A. Gude, S. C. Kao, K. Klapoetke, Y. Kubota, J. Mans, N. Pastika, R. Rusack, A. Singovsky, N. Tambe, J. Turkewitz, J. G. Acosta, S. Oliveros, E. Avdeeva, K. Bloom, S. Bose, D. R. Claes, A. Dominguez, R. Gonzalez Suarez, J. Keller, D. Knowlton, I. Kravchenko, J. Lazo-Flores, F. Meier, F. Ratnikov, G. R. Snow, M. Zvada, J. Dolen, A. Godshalk, I. Iashvili, A. Kharchilava, A. Kumar, S. Rappoccio, G. Alverson, E. Barberis, D. Baumgartel, M. Chasco, A. Massironi, D. M. Morse, D. Nash, T. Orimoto, D. Trocino, R. J. Wang, D. Wood, J. Zhang, K. A. Hahn, A. Kubik, N. Mucia, N. Odell, B. Pollack, A. Pozdnyakov, M. Schmitt, S. Stoynev, K. Sung, M. Velasco, S. Won, A. Brinkerhoff, K. M. Chan, A. Drozdetskiy, M. Hildreth, C. Jessop, D. J. Karmgard, N. Kellams, K. Lannon, S. Lynch, N. Marinelli, Y. Musienko, T. Pearson, M. Planer, R. Ruchti, G. Smith, N. Valls, M. Wayne, M. Wolf, A. Woodard, L. Antonelli, J. Brinson, B. Bylsma, L. S. Durkin, S. Flowers, A. Hart, C. Hill, R. Hughes, K. Kotov, T. Y. Ling, W. Luo, D. Puigh, M. Rodenburg, B. L. Winer, H. Wolfe, H. W. Wulsin, O. Driga, P. Elmer, J. Hardenbrook, P. Hebda, A. Hunt, S. A. Koay, P. Lujan, D. Marlow, T. Medvedeva, M. Mooney, J. Olsen, P. Piroué, X. Quan, H. Saka, D. Stickland, C. Tully, J. S. Werner, A. Zuranski, E. Brownson, S. Malik, H. Mendez, J. E. Ramirez Vargas, V. E. Barnes, D. Benedetti, D. Bortoletto, M. De Mattia, L. Gutay, Z. Hu, M. K. Jha, M. Jones, K. Jung, M. Kress, N. Leonardo, D. H. Miller, N. Neumeister, B. C. Radburn-Smith, X. Shi, I. Shipsey, D. Silvers, A. Svyatkovskiy, F. Wang, W. Xie, L. Xu, J. Zablocki, N. Parashar, J. Stupak, A. Adair, B. Akgun, K. M. Ecklund, F. J. M. Geurts, W. Li, B. Michlin, B. P. Padley, R. Redjimi, J. Roberts, J. Zabel, B. Betchart, A. Bodek, R. Covarelli, P. de Barbaro, R. Demina, Y. Eshaq, T. Ferbel, A. Garcia-Bellido, P. Goldenzweig, J. Han, A. Harel, A. Khukhunaishvili, S. Korjenevski, G. Petrillo, D. Vishnevskiy, R. Ciesielski, L. Demortier, K. Goulianos, C. Mesropian, S. Arora, A. Barker, J. P. Chou, C. Contreras-Campana, E. Contreras-Campana, D. Duggan, D. Ferencek, Y. Gershtein, R. Gray, E. Halkiadakis, D. Hidas, S. Kaplan, A. Lath, S. Panwalkar, M. Park, R. Patel, S. Salur, S. Schnetzer, S. Somalwar, R. Stone, S. Thomas, P. Thomassen, M. Walker, K. Rose, S. Spanier, A. York, O. Bouhali, A. Castaneda Hernandez, R. Eusebi, W. Flanagan, J. Gilmore, T. Kamon, V. Khotilovich, V. Krutelyov, R. Montalvo, I. Osipenkov, Y. Pakhotin, A. Perloff, J. Roe, A. Rose, A. Safonov, I. Suarez, A. Tatarinov, K. A. Ulmer, N. Akchurin, C. Cowden, J. Damgov, C. Dragoiu, P. R. Dudero, J. Faulkner, K. Kovitanggoon, S. Kunori, S. W. Lee, T. Libeiro, I. Volobouev, E. Appelt, A. G. Delannoy, S. Greene, A. Gurrola, W. Johns, C. Maguire, Y. Mao, A. Melo, M. Sharma, P. Sheldon, B. Snook, S. Tuo, J. Velkovska, M. W. Arenton, S. Boutle, B. Cox, B. Francis, J. Goodell, R. Hirosky, A. Ledovskoy, H. Li, C. Lin, C. Neu, J. Wood, C. Clarke, R. Harr, P. E. Karchin, C. Kottachchi Kankanamge Don, P. Lamichhane, J. Sturdy, D. A. Belknap, D. Carlsmith, M. Cepeda, S. Dasu, L. Dodd, S. Duric, E. Friis, R. Hall-Wilton, M. Herndon, A. Hervé, P. Klabbers, A. Lanaro, C. Lazaridis, A. Levine, R. Loveless, A. Mohapatra, I. Ojalvo, T. Perry, G. A. Pierro, G. Polese, I. Ross, T. Sarangi, A. Savin, W. H. Smith, D. Taylor, C. Vuosalo, N. Woods, CMS Collaboration

**Affiliations:** Yerevan Physics Institute, Yerevan, Armenia; Institut für Hochenergiephysik der OeAW, Vienna, Austria; National Centre for Particle and High Energy Physics, Minsk, Belarus; Universiteit Antwerpen, Antwerp, Belgium; Vrije Universiteit Brussel, Brussels, Belgium; Université Libre de Bruxelles, Brussels, Belgium; Ghent University, Ghent, Belgium; Université Catholique de Louvain, Louvain-la-Neuve, Belgium; Université de Mons, Mons, Belgium; Centro Brasileiro de Pesquisas Fisicas, Rio de Janeiro, Brazil; Universidade do Estado do Rio de Janeiro, Rio de Janeiro, Brazil; Universidade Estadual Paulista, Universidade Federal do ABC, São Paulo, Brazil; Institute for Nuclear Research and Nuclear Energy, Sofia, Bulgaria; University of Sofia, Sofia, Bulgaria; Institute of High Energy Physics, Beijing, China; State Key Laboratory of Nuclear Physics and Technology, Peking University, Beijing, China; Universidad de Los Andes, Bogotá, Colombia; Faculty of Electrical Engineering, Mechanical Engineering and Naval Architecture, University of Split, Split, Croatia; Faculty of Science, University of Split, Split, Croatia; Institute Rudjer Boskovic, Zagreb, Croatia; University of Cyprus, Nicosia, Cyprus; Charles University, Prague, Czech Republic; Academy of Scientific Research and Technology of the Arab Republic of Egypt, Egyptian Network of High Energy Physics, Cairo, Egypt; National Institute of Chemical Physics and Biophysics, Tallinn, Estonia; Department of Physics, University of Helsinki, Helsinki, Finland; Helsinki Institute of Physics, Helsinki, Finland; Lappeenranta University of Technology, Lappeenranta, Finland; DSM/IRFU, CEA/Saclay, Gif-sur-Yvette, France; Laboratoire Leprince-Ringuet, Ecole Polytechnique, IN2P3-CNRS, Palaiseau, France; Institut Pluridisciplinaire Hubert Curien, Université de Strasbourg, Université de Haute Alsace Mulhouse, CNRS/IN2P3, Strasbourg, France; Centre de Calcul de l’Institut National de Physique Nucleaire et de Physique des Particules, CNRS/IN2P3, Villeurbanne, France; Institut de Physique Nucléaire de Lyon, Université de Lyon, Université Claude Bernard Lyon 1, CNRS-IN2P3, Villeurbanne, France; Institute of High Energy Physics and Informatization, Tbilisi State University, Tbilisi, Georgia; I. Physikalisches Institut, RWTH Aachen University, Aachen, Germany; III. Physikalisches Institut A, RWTH Aachen University, Aachen, Germany; III. Physikalisches Institut B, RWTH Aachen University, Aachen, Germany; Deutsches Elektronen-Synchrotron, Hamburg, Germany; University of Hamburg, Hamburg, Germany; Institut für Experimentelle Kernphysik, Karlsruhe, Germany; Institute of Nuclear and Particle Physics (INPP), NCSR Demokritos, Aghia Paraskevi, Greece; University of Athens, Athens, Greece; University of Ioánnina, Ioannina, Greece; Wigner Research Centre for Physics, Budapest, Hungary; Institute of Nuclear Research ATOMKI, Debrecen, Hungary; University of Debrecen, Debrecen, Hungary; National Institute of Science Education and Research, Bhubaneswar, India; Panjab University, Chandigarh, India; University of Delhi, Delhi, India; Saha Institute of Nuclear Physics, Kolkata, India; Bhabha Atomic Research Centre, Mumbai, India; Tata Institute of Fundamental Research, Mumbai, India; Institute for Research in Fundamental Sciences (IPM), Tehran, Iran; University College Dublin, Dublin, Ireland; INFN Sezione di Bari, Università di Bari, Politecnico di Bari, Bari, Italy; INFN Sezione di Bologna, Università di Bologna, Bologna, Italy; INFN Sezione di Catania, Università di Catania, CSFNSM, Catania, Italy; INFN Sezione di Firenze, Università di Firenze, Florence, Italy; INFN Laboratori Nazionali di Frascati, Frascati, Italy; INFN Sezione di Genova, Università di Genova, Genoa, Italy; INFN Sezione di Milano-Bicocca, Università di Milano-Bicocca, Milan, Italy; INFN Sezione di Napoli, Università di Napoli ’Federico II’, Università della Basilicata (Potenza), Università G. Marconi (Roma), Naples, Italy; INFN Sezione di Padova, Università di Padova, Università di Trento (Trento), Padua, Italy; INFN Sezione di Pavia, Università di Pavia, Pavia, Italy; INFN Sezione di Perugia, Università di Perugia, Perugia, Italy; INFN Sezione di Pisa, Università di Pisa, Scuola Normale Superiore di Pisa, Pisa, Italy; INFN Sezione di Roma, Università di Roma, Rome, Italy; INFN Sezione di Torino, Università di Torino, Università del Piemonte Orientale (Novara), Turin, Italy; INFN Sezione di Trieste, Università di Trieste, Trieste, Italy; Kangwon National University, Chunchon, Korea; Kyungpook National University, Taegu, Korea; Chonbuk National University, Chonju, Korea; Chonnam National University, Institute for Universe and Elementary Particles, Kwangju, Korea; Korea University, Seoul, Korea; Seoul National University, Seoul, Korea; University of Seoul, Seoul, Korea; Sungkyunkwan University, Suwon, Korea; Vilnius University, Vilnius, Lithuania; National Centre for Particle Physics, Universiti Malaya, Kuala Lumpur, Malaysia; Centro de Investigacion y de Estudios Avanzados del IPN, Mexico City, Mexico; Universidad Iberoamericana, Mexico City, Mexico; Benemerita Universidad Autonoma de Puebla, Puebla, Mexico; Universidad Autónoma de San Luis Potosí, San Luis Potosí, Mexico; University of Auckland, Auckland, New Zealand; University of Canterbury, Christchurch, New Zealand; National Centre for Physics, Quaid-I-Azam University, Islamabad, Pakistan; National Centre for Nuclear Research, Swierk, Poland; Institute of Experimental Physics, Faculty of Physics, University of Warsaw, Warsaw, Poland; Laboratório de Instrumentação e Física Experimental de Partículas, Lisbon, Portugal; Joint Institute for Nuclear Research, Dubna, Russia; Petersburg Nuclear Physics Institute, Gatchina, St. Petersburg, Russia; Institute for Nuclear Research, Moscow, Russia; Institute for Theoretical and Experimental Physics, Moscow, Russia; P. N. Lebedev Physical Institute, Moscow, Russia; Skobeltsyn Institute of Nuclear Physics, Lomonosov Moscow State University, Moscow, Russia; State Research Center of Russian Federation, Institute for High Energy Physics, Protvino, Russia; Faculty of Physics, Vinca Institute of Nuclear Sciences, University of Belgrade, Belgrade, Serbia; Centro de Investigaciones Energéticas Medioambientales y Tecnológicas (CIEMAT), Madrid, Spain; Universidad Autónoma de Madrid, Madrid, Spain; Universidad de Oviedo, Oviedo, Spain; Instituto de Física de Cantabria (IFCA), CSIC-Universidad de Cantabria, Santander, Spain; CERN, European Organization for Nuclear Research, Geneva, Switzerland; Paul Scherrer Institut, Villigen, Switzerland; Institute for Particle Physics, ETH Zurich, Zurich, Switzerland; Universität Zürich, Zurich, Switzerland; National Central University, Chung-Li, Taiwan; National Taiwan University (NTU), Taipei, Taiwan; Department of Physics, Faculty of Science, Chulalongkorn University, Bangkok, Thailand; Cukurova University, Adana, Turkey; Physics Department, Middle East Technical University, Ankara, Turkey; Bogazici University, Istanbul, Turkey; Istanbul Technical University, Istanbul, Turkey; National Scientific Center, Kharkov Institute of Physics and Technology, Kharkov, Ukraine; University of Bristol, Bristol, UK; Rutherford Appleton Laboratory, Didcot, UK; Imperial College, London, UK; Brunel University, Uxbridge, UK; Baylor University, Waco, USA; The University of Alabama, Tuscaloosa, USA; Boston University, Boston, USA; Brown University, Providence, USA; University of California, Davis, USA; University of California, Los Angeles, USA; University of California, Riverside, Riverside, USA; University of California, San Diego, La Jolla, USA; University of California, Santa Barbara, Santa Barbara, USA; California Institute of Technology, Pasadena, USA; Carnegie Mellon University, Pittsburgh, USA; University of Colorado at Boulder, Boulder, USA; Cornell University, Ithaca, USA; Fairfield University, Fairfield, USA; Fermi National Accelerator Laboratory, Batavia, USA; University of Florida, Gainesville, USA; Florida International University, Miami, USA; Florida State University, Tallahassee, USA; Florida Institute of Technology, Melbourne, USA; University of Illinois at Chicago (UIC), Chicago, USA; The University of Iowa, Iowa City, USA; Johns Hopkins University, Baltimore, USA; The University of Kansas, Lawrence, USA; Kansas State University, Manhattan, USA; Lawrence Livermore National Laboratory, Livermore, USA; University of Maryland, College Park, USA; Massachusetts Institute of Technology, Cambridge, USA; University of Minnesota, Minneapolis, USA; University of Mississippi, Oxford, USA; University of Nebraska-Lincoln, Lincoln, USA; State University of New York at Buffalo, Buffalo, USA; Northeastern University, Boston, USA; Northwestern University, Evanston, USA; University of Notre Dame, Notre Dame, USA; The Ohio State University, Columbus, USA; Princeton University, Princeton, USA; University of Puerto Rico, Mayagüez, USA; Purdue University, West Lafayette, USA; Purdue University Calumet, Hammond, USA; Rice University, Houston, USA; University of Rochester, Rochester, USA; The Rockefeller University, New York, USA; Rutgers, The State University of New Jersey, Piscataway, USA; University of Tennessee, Knoxville, USA; Texas A&M University, College Station, USA; Texas Tech University, Lubbock, USA; Vanderbilt University, Nashville, USA; University of Virginia, Charlottesville, USA; Wayne State University, Detroit, USA; University of Wisconsin, Madison, USA; CERN, 1211 Geneva 23, Switzerland

## Abstract

This paper presents distributions of topological observables in inclusive three- and four-jet events produced in pp collisions at a centre-of-mass energy of 7$\,\text {TeV}$ with a data sample collected by the CMS experiment corresponding to a luminosity of 5.1$\,\text {fb}^{-1}$. The distributions are corrected for detector effects, and compared with several event generators based on two- and multi-parton matrix elements at leading order. Among the considered calculations, MadGraph interfaced with pythia6 displays the overall best agreement with data.

## Introduction

In proton-proton collisions at the LHC, interactions take place between the partons of the colliding protons. The scattered partons from hard collisions fragment and hadronize into collimated groups of particles called jets. The study of jets with high transverse momentum ($p_{\mathrm {T}}$) provides a test of the predictions from quantum chromodynamics (QCD) and deviations from these predictions can be used to look for physics beyond the standard model. While parton scattering is an elementary QCD process that can be calculated from first principles, predictions of jet distributions require an accurate hadronization model. In this paper, several hadronization models are examined.

High-$p_{\mathrm {T}}$ parton production is described by perturbative QCD (pQCD) in terms of the scattering cross section convolved with a parton distribution function (PDF) for each parton that parametrizes the momentum distribution of partons within the proton. The hard-scattering cross section itself can be written as an expansion in the strong coupling constant $\alpha _{\mathrm {s}}$. The leading term in this expansion corresponds to the emission of two partons. The next term includes diagrams where an additional parton is present in the final state as a result of hard-gluon radiation (e.g. $\mathrm{g} \mathrm{g} \rightarrow \mathrm{g} \mathrm{g} \mathrm{g}$). Cross sections for such processes diverge when any of the three partons becomes soft or when two of the partons become collinear. Finally, pQCD predicts three classes of four-jet events that correspond to the processes $\mathrm{q} \mathrm{q}/\mathrm{g} \mathrm{g} \rightarrow \mathrm{q} \mathrm{q} \mathrm{g} \mathrm{g}, \mathrm{q} \mathrm{q}/\mathrm{g} \mathrm{g} \rightarrow \mathrm{q} \mathrm{q} \mathrm{q} \mathrm{q}$ and $\mathrm{q} \mathrm{g} \rightarrow \mathrm{q} \mathrm{g} \mathrm{g} \mathrm{g}/\mathrm{q} \mathrm{q} \mathrm{q} \mathrm{g}$, where $\mathrm{q}$ stands for both quarks and anti-quarks. Processes with two or more gluons in the final state receive a contribution from the triple-gluon vertex, a consequence of the non-Abelian structure of QCD.

We are studying distributions of topological variables, which are sensitive to QCD color factors, the spin structure of gluons, and hadronization models. These topological variables were studied widely in the earlier LEP [1, 2] and the Tevatron [3, 4] experiments and help to validate theoretical models implemented in various Monte Carlo (MC) event generators.

The distributions of multijet variables are sensitive to the treatment of the higher-order processes and approximations involved. Many MC event generators make use of leading order (LO) matrix elements (ME) in the primary 2 $\rightarrow$ 2 process. A good agreement between the measurements and MC predictions can establish the validity of the treatment of higher-order effects, and any large deviation may lead to large systematic uncertainties in searches for new physics.

The multijet observables presented here are based on hadronic events from 7$\,\text {TeV}$ pp collision data recorded with the CMS detector corresponding to an integrated luminosity of 5.1$\,\text {fb}^{-1}$. The kinematic and angular properties of these events are computed from the jet momentum four-vectors. Unfolding techniques are used to correct for the effects of the detector resolution and efficiency. Systematic uncertainties resulting from the limited knowledge of the jet energy scale (JES), jet energy and angular resolution (JER), unfolding, and event selection are estimated, and the unfolded distributions are compared with predictions of several QCD-based MC models.

In this paper, the CMS detector is briefly described in Sect. 2. Sections 3 and 4 summarize the MC models used and the variables studied in this paper. Event selection and measurements are described in Sects. 5 and 6, respectively. The correction of the distributions due to detector effects is discussed in Sect. . Sections 8 and 9 describe the estimation of systematic uncertainties and the final results. The overall summary is given in Sect. 10.

## The CMS detector

The central feature of the CMS apparatus is a superconducting solenoid of 6$\text {\,m}$ internal diameter, providing a magnetic field of 3.8$\text {\,T}$. Within the field volume are a silicon pixel and strip tracker, a lead tungstate crystal electromagnetic calorimeter (ECAL), and a brass and scintillator hadron calorimeter (HCAL), each composed of a barrel and two endcap sections. Muons are measured in gas-ionization detectors embedded in the steel flux-return yoke outside the solenoid. Extensive forward calorimetry complements the coverage provided by the barrel and endcap detectors. The barrel and endcap calorimeters cover a pseudorapidity region $-3.0<\eta <3.0$. Pseudorapidity is defined as $\eta = - \ln \tan [\theta /2]$, where $\theta$ is the polar angle. The transition between barrel and endcaps happens at $|\eta | = 1.479$ for the ECAL and $|\eta |= 1.15$ for the HCAL. The first level (L1) of the CMS trigger system, composed of custom hardware processors, uses information from the calorimeters and muon detectors to select the most interesting events in a fixed time interval of less than 4$\,\mu \text {s}$. The high-level trigger (HLT) processor farm further decreases the event rate from around 100$\text {\,kHz}$ to around 400$\text {\,Hz}$ before data storage. A more detailed description of the CMS detector, together with a definition of the coordinate system used and the relevant kinematic variables, can be found in Ref. [5].

## Monte Carlo models

The MC event generators rely on models using modified LO QCD calculations. The elementary hard process between the partons is computed at LO. The parton shower (PS), used to simulate higher-order processes, follows an ordering principle motivated by QCD. Nevertheless, the parton shower models can differ in the ordering of emissions and the event generators can also have different treatments of beam remnants and multiple interactions.

The pythia 6.4.26 [6] event generator uses a PS model to simulate higher-order processes [7–9] after the LO ME from pQCD calculations. The PS model, ordered by the $p_{\mathrm {T}}$ of the emissions, provides a good description of event shapes when the emitted partons are close in phase space. Events are generated with the Z2 tune [10] for the underlying event. This tune is identical to the Z1 tune [11], except that it uses CTEQ6L1 [12] PDFs. The partons are hadronized (process of converting the partons into measured particles) using the Lund string model [13, 14].

The pythia 8.153 [15] event generator also uses a PS model with the successive emissions of partons ordered in $p_{\mathrm {T}}$ and the Lund string model for hadronization. The main difference between the two pythia versions is the description of multiparton interactions (MPI). In pythia 8, initial state radiation (ISR), final state radiation (FSR), and MPI are interleaved in the $p_{\mathrm {T}}$ ordering, while in pythia 6, only ISR and FSR are interleaved. The tune 4C [16] is used with this generator. This tune uses CTEQ6L1 PDFs with parameters using CDF as well as early LHC measurements.

The herwig++ 2.4.2 [17] tune 23 [18] program takes the LO ME and simulates a PS using the coherent branching algorithm with angular ordering [19] of the showers. The partons are hadronized in this model using a cluster model [20] and the underlying event is simulated using the eikonal multiple partonic scattering model.

In the case of MadGraph 5.1.5.7 [21], multiparton final states are also computed at tree level. The parton shower and nonperturbative parts for Madgraph 5.1.5.7 simulation sample is handled by pythia 6.4.26 with Z2 tune. The MLM matching procedure [22] is used to avoid double counting between the ME and the PS. The MadGraph samples are created in four bins of the variable $H_{\mathrm {T}}$, the scalar sum of the parton $p_{\mathrm {T}}$. The matching between ME and PS has been studied in detail and has been validated using inclusive jet $p_{\mathrm {T}}$ distributions. Several samples are generated using different matching parameters and are used in estimating systematic uncertainty in the theoretical prediction.

These MC programs are the most commonly used models to describe multi-partonic final states and are normally used to describe QCD background in searches within CMS. The events produced from these models are simulated using a CMS detector simulation program based on Geant4 [23] and reconstructed with the same program used for the data. These MC events are used for the comparison with the measurements as well as to correct the distributions for detector effects.

## Definition of variables

### Three-jet variables

The topological variables used in this study are defined in the parton or jet centre-of-mass (CM) system. The topological properties of the three-parton final state in the CM system can be described in terms of five variables [3]. Three of the variables reflect partition of the CM energy among the three final-state partons. There are three angles, which define the spatial orientation in the plane containing the three partons, but only two are independent.

**Fig. 1:**
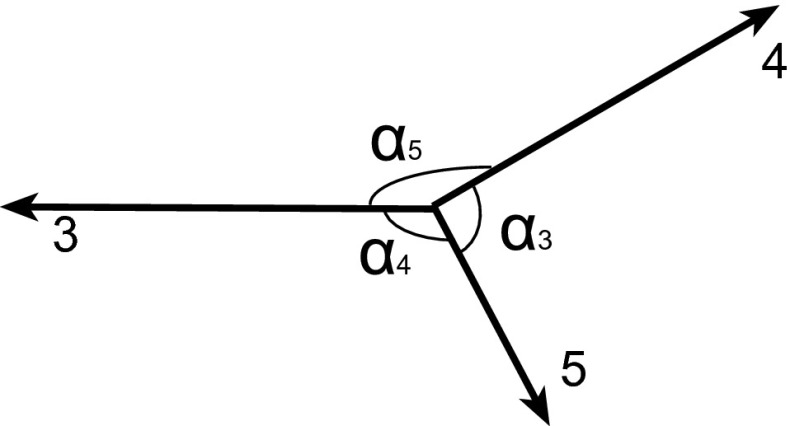
Illustration of the three-jet variables in the process 1+2$\rightarrow$3+4+5. The scaled energies are related to the angles ($\alpha _i$) among the jets for massless parton

It is convenient to introduce the notation $1+2\rightarrow 3+4+5$ for the three-parton process. Here, numbers 1 and 2 refer to incoming partons while the numbers 3, 4, and 5 label the outgoing partons in a descending order in energies in the parton CM frame, i.e. $E_3>E_4>E_5$ (Fig. 1). The final-state parton energy is an obvious choice for the topological variable for the three-parton final state. For simplicity, $E_i$ ($i = 3$, 4, 5) is often replaced by the scaled variable $x_i$ ($i = 3$, 4, 5), which is defined by $x_i = 2E_i/\sqrt{\hat{s}_{345}}$, where $\sqrt{\hat{s}_{345}}$ is the CM energy of the hard-scattering process. It is also referred to as the mass of the three-parton system, and by definition,

1 $$\begin{aligned} x_3 + x_4 + x_5 = 2. \end{aligned}$$

The internal structure of the three-parton final state is determined by any two scaled parton energies. The third one is calculated using Eq. 1. It needs two angular variables which fix the event orientation. In total, five independent kinematic variables are needed to describe the topological properties of the three-parton final state. In this analysis, however, the study is restricted to three variables: $\sqrt{{\hat{s}_{345}}}$, $x_3$, and $x_4$, while the angular variables are not included.

### Four-jet variables

To define a four-parton final state in its CM frame, eight independent parameters are needed. Two of these define the overall event orientation, while the other six fix the internal structure of the four-parton system. In contrast to the three-parton final state, there is no simple relationship between the scaled parton energies and the opening angles between partons. Consequently, the choice of topological variables is less obvious in this case. Variables are defined here in a way similar to those investigated for the three-parton final state. The four partons are ordered in descending energy in the parton CM frame and labeled from 3 to 6. The variables include the scaled energies and the polar angles of the four partons with respect to the beams.

In addition to the four-parton CM energy or the mass of the four-parton system ($\sqrt{\hat{s}_{3456}}$), two angular distributions characterizing the orientation of event planes are investigated. One of these is the Bengtsson–Zerwas angle ($\chi _{\mathrm {BZ}}$) [24] defined as the angle between the plane containing the two leading jets and the plane containing the two nonleading jets:

2 $$\begin{aligned} \cos \chi _{\mathrm {BZ}} = \frac{(\mathbf {p}_3\times \mathbf {p}_4)\cdot (\mathbf {p}_5\times \mathbf {p}_6)}{|\mathbf {p}_3\times \mathbf {p}_4 ||\mathbf {p}_5\times \mathbf {p}_6 |}. \end{aligned}$$

The second variable is the cosine of the Nachtmann–Reiter angle ($\cos \theta _{\mathrm {NR}}$) [25] defined as the angle between the momentum vector differences of the two leading jets and the two nonleading jets:

3 $$\begin{aligned} \cos \theta _{\mathrm {NR}} = \frac{(\mathbf {p}_3 - \mathbf {p}_4)\cdot (\mathbf {p}_5 - \mathbf {p}_6)}{|\mathbf {p}_3 - \mathbf {p}_4 ||\mathbf {p}_5 - \mathbf {p}_6 |}. \end{aligned}$$

**Fig. 2:**
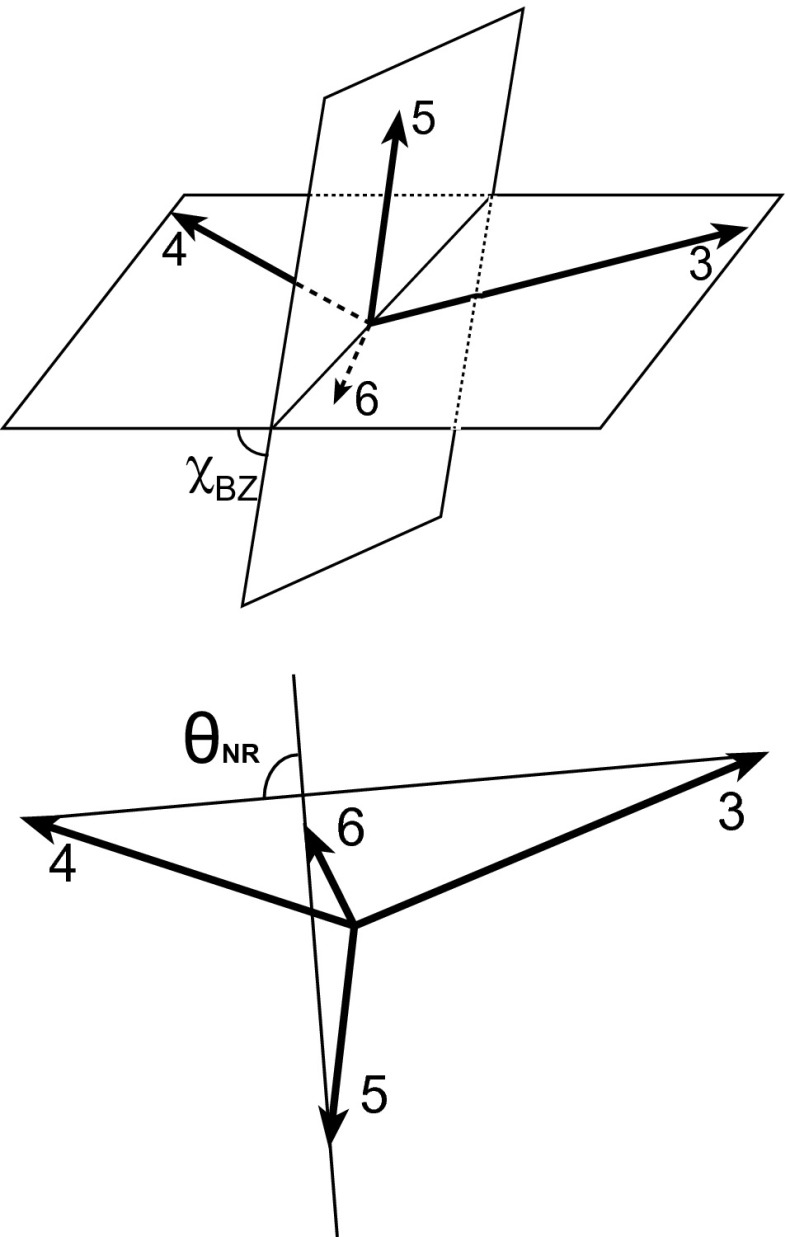
Illustration of the Bengtsson–Zerwas angle ($\chi _{\mathrm {BZ}}$) and the Nachtmann–Reiter angle ($\theta _{\mathrm {NR}}$) definitions for the four-jet events. The *top* *figure* shows the Bengtsson–Zerwas angle, which is the angle between the plane containing the two leading jets and the plane containing the two nonleading jets. The *bottom* *figure* shows the Nachtmann–Reiter angle, which is the angle between the momentum vector differences of the two leading jets and the two nonleading jets

Figure 2 illustrates the definitions of $\chi _{\mathrm {BZ}}$ and $\theta _{\mathrm {NR}}$ variables. Historically, $\chi _{\mathrm {BZ}}$ and $\theta _{\mathrm {NR}}$ were proposed for $\mathrm {e}^+\mathrm {e}^-$ collisions to study gluon self-coupling. Their interpretation in pp collisions is more complicated, but the variables can be used as a tool for studying the internal structure of the four-jet events.

## Data samples and event selection

Jets are reconstructed from particle-flow (PF) objects [26, 27] using the anti-$k_{\mathrm {T}}$ clustering algorithm [28] with the distance parameter $R = 0.5$, as calculated with Fastjet 2.0 [29]. The PF algorithm utilizes the best energy measurements of each particle candidate from the most suitable combination of the detector components. A cluster is formed from all the particle-flow candidates that satisfy the chosen distance parameter. The four-momentum of the jet is then defined as the sum of four-momenta of the corresponding particle-flow candidates, which results in jets with nonzero mass.

The JES correction applied to jets used in this analysis is based on high-$p_{\mathrm {T}}$ jet events generated by pythia 6 and then simulated using Geant4, and in situ measurements with dijet and photon $+$ jet events [30]. An average of ten minimum bias interactions occur in each pp bunch crossing (pileup), and this requires an additional correction to remove the extra energy deposited by these pileup events. The size of the correction depends on the $p_{\mathrm {T}}$ and $\eta$ of the jet. The correction appears as a multiplicative factor to the jet energy, and is typically less than 1.2 and approximately uniform in $\eta$.

Events passing single-jet HLT requirements are used in this analysis. These triggers require jets reconstructed from calorimetric information with the anti-$k_{\mathrm {T}}$ clustering algorithm and with energy corrections applied. Jets are ordered in decreasing jet $p_{\mathrm {T}}$, and the leading jet $p_{\mathrm {T}}$ is required to be above a certain threshold. As offline jets are reconstructed with the PF algorithm, this may result in a trigger not being fully efficient near the threshold. Trigger efficiencies are studied as a function of the leading jet $p_{\mathrm {T}}$ for all trigger thresholds. Values of the leading jet $p_{\mathrm {T}}$, where the trigger efficiency is determined to be larger than 99 %, are listed in Table 1. It also summarizes the prescale factors and the effective integrated luminosities collected using the different HLT thresholds.

**Table 1:** Prescales, integrated luminosity and offline $p_{\mathrm {T}}$ threshold of the leading jet for different trigger paths. The terminology for Level 1 (L1) triggers as well as HLT includes the jet $p_{\mathrm {T}}$ threshold (in $\,\text {GeV}$) applicable to the trigger

Period	HLT L1	HLT60 SingleJet36	HLT110 SingleJet68	HLT190 SingleJet92	HLT240 SingleJet92	HLT370 SingleJet92/SingleJet128
2011A	L1 prescale	1–300	1–10	1	1	1
	HLT prescale	15–180	1–5000	1–60	1–24	1
	$\int {\mathcal {L}}$ (pb^−1^)	0.29	6.16	114.7	392.2	2328
2011B	L1 prescale	50–400	1–20	1–10	1	1
	HLT prescale	80–84	80–1000	10–100	4–30	1
	$\int {\mathcal {L}}$ (pb^−1^)	0.12	1.12	40.2	136.0	2767
Overall	$\int {\mathcal {L}}$ (pb^−1^)	0.41	7.29	154.8	528.2	5096
	$p_{\mathrm {T}}$ threshold	110$\,\text {GeV}$	190$\,\text {GeV}$	300$\,\text {GeV}$	360$\,\text {GeV}$	500$\,\text {GeV}$

Jets are selected with restrictive criteria on the neutral energy fractions (both electromagnetic and hadronic components), and all the jets are required to have $$p_{\mathrm {T}} > 50$$$$\,\text {GeV}$$ and absolute rapidity, ($$y = (1/2)\ln [(E+p_z)/(E-p_z)]$$), $$|y |\le 2.5$$. The jet with the highest $$p_{\mathrm {T}}$$ is required to be above a threshold as given by the requirement from the trigger turn-on curve. To avoid overlap of events from two different HLT paths, the $$p_{\mathrm {T}}$$ of the leading jet is also required to be less than an upper value. The overall criteria are summarized in Table 2. Though data from all the five trigger paths are studied, figures from two representative trigger paths (the highest $$p_{\mathrm {T}}$$ threshold and a lower one with good statistical accuracy) are presented in this paper.

**Table 2:** Threshold of the leading jet $p_{\mathrm {T}}$ for different HLT paths. This paper shows results from two representative trigger paths HLT110 and HLT370

HLT	HLT60	HLT110	HLT190	HLT240	HLT370
Leading jet $p_{\mathrm {T}}$ ($\text {GeV}$ )	110–190	190–300	300–360	360–500	$>$500

Events are selected with at least three jets passing the selection criteria as stated above. Additional selection requirements are also applied to reduce backgrounds due to beam halo, cosmic rays and detector noise. The event must have at least one good reconstructed vertex [31]. Missing transverse energy, $E_{\mathrm {T}}^{\text {miss}}$, is required to be less than $0.3 \sum E_{\mathrm {T}}$, where the summation is over all PF jets. The quantities $E_{\mathrm {T}}^{\text {miss}}$ and $\sum E_{\mathrm {T}}$ are obtained from negative vector sum and scalar sum of the transverse momenta of the jets, respectively. A number of event filters [32] accept only those events that have negligible noise in the detector. The jets are ordered in decreasing $p_{\mathrm {T}}$, and an event with at least three (four) jets satisfying the jet selection criteria is classified as a three-jet (four-jet) event.

## Measurements

The 4-momenta of all the jets in the three- or four-jet event category are transformed into the CM frame of the three- or four-jet system. The jets are then ordered in decreasing energy. The three- and four-jet variables as described in Sect. 4 are then calculated from the kinematic and angular information of the jets. Since detector resolution varies over the potential kinematic ranges, variable bin widths are adopted for the jet masses and the scaled jet energies, while for angular variables constant bin widths are used.

### Detector-level distributions

The measured distributions of the three- and four-jet variables are compared with predictions from two MC generators (pythia 6 and MadGraph  $+$ pythia 6), simulated using the identical detector condition as that in the data. The identical pileup condition is obtained by reweighting the MC simulation to match the spectrum of pileup interactions observed in the data. The size of the reweighting correction is typically less than 1 %. The agreement between the data and the MC predictions is reasonable, so these MC generators are used to correct the measured distributions.

**Fig. 3:**
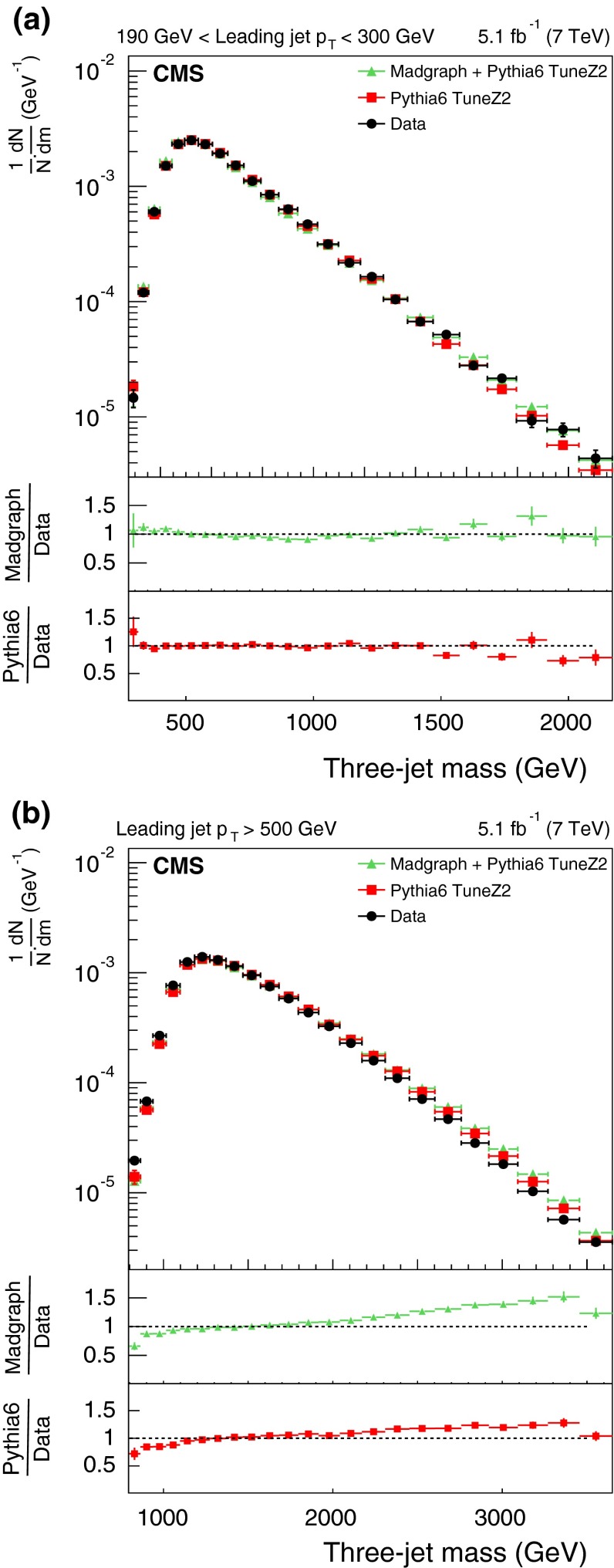
The *upper panels* display the normalized distributions of the reconstructed three-jet mass for events where the most forward jet has $|y |<2.5$. *Figures* differ by $p_{\mathrm {T}}$ ranges of the leading jet: 190–300$\,\text {GeV}$ (**a**), and above 500$\,\text {GeV}$ (**b**) for data (before correction due to detector effects) and predictions from MC generators. The *bottom panel of each plot* shows the ratio of MC predictions to the data. The data are shown with only statistical uncertainty

**Fig. 4:**
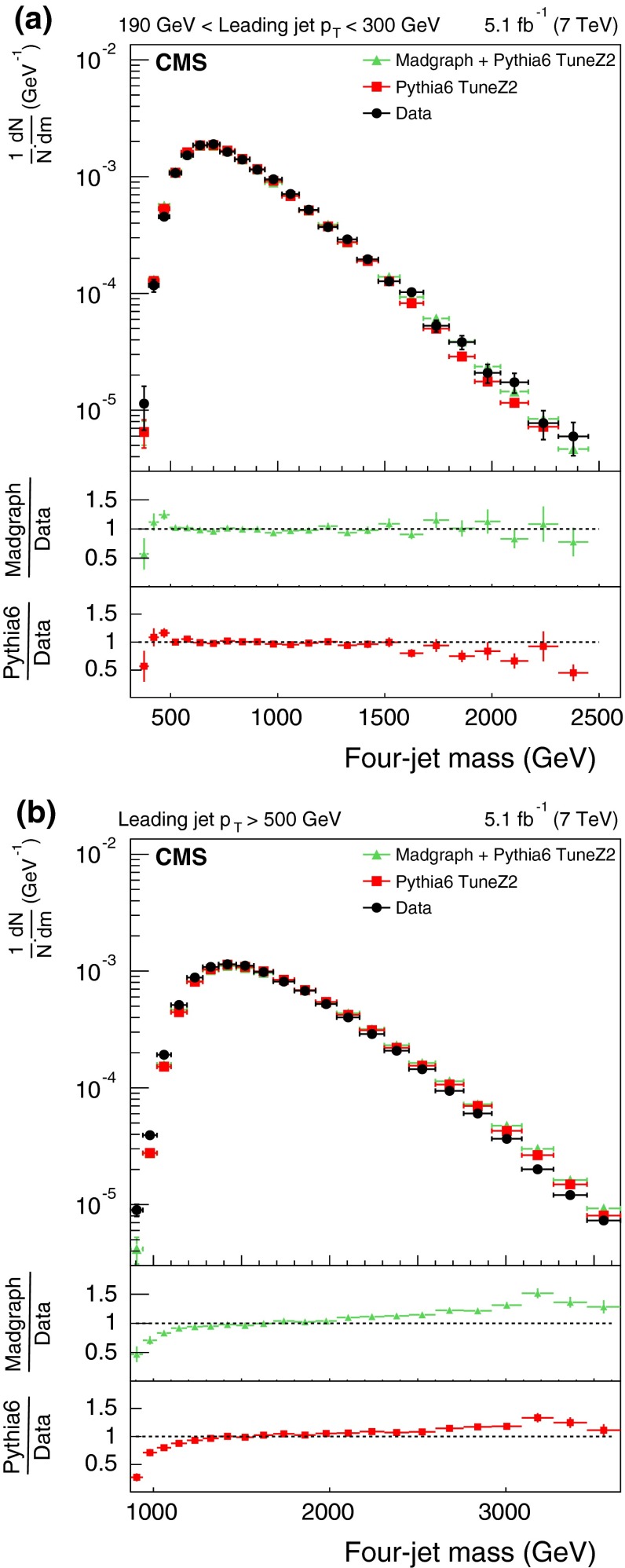
The *upper panels* display the normalized distributions of the reconstructed four-jet mass for events where the most forward jet has $|y |< 2.5$. The other explanations are the same as Fig. 3

Figures 3 and 4 show the normalized three- and four-jet mass distributions. The data are compared with two different MC programs: pythia 6 and MadGraph  $+$ pythia 6, each with two different HLTs with $p_{\mathrm {T}}$ thresholds above 110 and 370$\,\text {GeV}$. As can be seen from the figures, there is agreement within a few percent between the data and the predictions of these two simulations. The difference between the predictions and the data varies typically from 4 to 10 %. However, there is a systematic deviation observed at high masses where the simulations are higher than the data.

## Corrections for detector effects

Multijet variables obtained from MC samples may differ from data because of the detector resolution and acceptance. Before comparisons with other experiments or theoretical predictions can be made, detector effects are unfolded into distributions at the final-state particle level. The basic component of the unfolding is the response function, where experimental observables are expressed as a function of theoretical observables. For simplicity, observables are taken in discrete sets, and the response function is replaced by a response matrix. The observed distribution is then unfolded with the inverse of response matrix to obtain a distribution corrected for detector effects. Matrix inversion has potential complications, because it cannot handle large statistical fluctuations and the matrix itself could be singular. Instead, we use the RooUnfold package [33] with the D’Agostini iterative method [34] as the default algorithm and the singular value decomposition method [35] for cross-checks.

The default response matrix is obtained using the pythia 6 event generator. Statistical uncertainties are estimated from the square root of the covariance matrix obtained from a variation of the results generated by simulated experiments.

The corrections for detector resolution and acceptance change the shape of the three-jet mass distributions by approximately 10 %, less than 5 % for the scaled energy of nonleading jets, and up to 20 % for the scaled energy of the leading jet. For four-jet variables, corrections applied are of the order of 20 % for the four-jet mass, 10 % for $\chi _{\mathrm {BZ}}$, and less than 5 % for cos $\theta _{\mathrm {NR}}$.

## Systematic uncertainties

The leading sources of systematic uncertainty are due to the JES, the JER, and the model dependence of corrections to the data. The distributions are presented in this analysis as normalized distributions, thus the absolute scale uncertainty of energy measurement does not play a significant role. There are insignificant contributions due to resolution of *y*. The main contribution of JES or JER to the uncertainty in the measurements is due to the migration of events from one category of jet multiplicity to the other.

The effect of pileup in the measured distributions has been studied as a function of the number of reconstructed vertices in the event. None of the variables show any significant dependence on the pileup condition, so systematic uncertainty due to pileup can be neglected.

### Jet energy scale

One of the dominant sources of systematic uncertainty is due to the jet energy scale corrections. The JES uncertainty has been estimated to be 2–2.5 % for PF jets [30], depending on the jet $p_{\mathrm {T}}$ and $\eta$. In order to map this uncertainty to the multijet variables, all jets in the selected events are systematically shifted by the respective uncertainties, and a new set of values for the multijet variables is calculated. This causes a migration of events from an event category of a given jet multiplicity to a different jet multiplicity. The migration could be as high as 20 % for some of the event categories. The corresponding distributions are then unfolded using the standard procedure as described in Sect. 7. The difference of these values from the central unfolded results is a measure of the uncertainty owing to the JES.

Uncertainties owing to the JES are found to be between 0.2–5.5 % in the three-jet mass, and 0.3–10 % in the four-jet mass. The systematic uncertainties are the largest at both ends of the mass spectra. The systematic uncertainties in scaled energy are between 0.1 and 2.0 %, and those in angular variables are in the range 0.1–3.0 %. There is a small increase in the uncertainty for distributions where there is at least one jet in the endcap region of the detector.

### Jet energy resolution

The JER is measured in data using the $p_{\mathrm {T}}$ balance in dijet events [36]. Based on these measurements, the resolution effects are corrected using simulated events. To study the effect of the difference between the simulated and the measured resolution, several sets of unfolded distributions are obtained using response matrices from the default resolution matrix and changing the jet resolution within its estimated uncertainty. Alternatively, the response matrix is constructed by convolving the generator level distribution with the measured resolution. The measured distribution is unfolded by this response matrix vis-a-vis the response matrix determined using fully simulated sample of pythia 6 events. These two estimates provide independent descriptions of the detector modeling and the difference is used as a measure of the systematic uncertainty due to detector performance. Position resolution affects the measurement of the jet direction, and it is estimated using simulated multijet events and validated with data.

Uncertainties owing to the JER are found to be between 0.1–10 % in the three-jet mass, 0.3–15 % in the four-jet mass, 0.1–10 % in the scaled jet energies and 0.2–8.2 % in the angular variables.

### Model dependence in unfolding

Unfolded distributions are obtained using two different response matrices derived from pythia 6 and MadGraph+pythia6 simulations. The difference in the unfolded values, due to the choice of response functions, gives a measure of the systematic uncertainty. The uncertainties are at the level of 0.1–6.0 % in the three-jet mass, 0.1–3.0 % in the scaled energy distributions of the three-jet variables, 0.1–8.0 % in the four-jet mass and 0.1–6.2 % in the angular variables in the four-jet samples. The uncertainties in the scaled jet energy increases by a few percent for the samples with lower values of leading jet $p_{\mathrm {T}}$.

Unfolding has been carried out using pythia 6 and MadGraph  $+$ pythia 6 samples, which has the same hadronization model. To test the effects of different hadronization models, MC samples from herwig++, which provides a different PS and hadronization approach, are used. However, the simulated event sample generated using herwig++ is statistically inadequate to be used in a complete unfolding procedure. The difference between bin-by-bin correction factors obtained with pythia 6 and herwig++ is found to be somewhat larger than the uncertainty due to the difference in the unfolding matrices: 0.1–12 % in the scaled energy distributions of three-jet variables, 0.1–7.7 % in the angular variables in the four-jet samples and 0.1–11.6 % in the jet masses. The larger values from the two estimates are chosen as the systematic uncertainties due to unfolding.

### Event selection

Jet candidates are required to pass certain criteria [37] designed to reduce unwanted detector effects. This analysis uses jets identified with very restrictive criteria on the ratio of the energy carried by neutral to that carried by charged particles. The effect of using these criteria is tested by reevaluating the same distributions with jets selected after relaxing the selection on the fractions of the energy carried by the neutral and the charged particles. Also, the selection on $E_{\mathrm {T}}^{\text {miss}}$ is changed, and the effect of this is estimated from the difference in the observed distributions. The uncertainty due to the event selection is found to be below 0.2 %.

### Overall uncertainty

The first three sources mentioned above are the dominant sources of systematic uncertainty. The contributions to the uncertainty from the selection requirements and pileup effects are found to be negligible. The uncertainties are calculated for each bin of the measured distributions and are added in quadrature. The overall systematic uncertainty is found to be smaller than the statistical uncertainty for most of the bins. Typical uncertainties for the six variables studied in this analysis are summarized in Table 3.

**Table 3:** Uncertainty ranges among the different bins in the topological distributions of the three- and four-jet variables

Uncertainty source	Uncertainty (%) for leading jet $p_{\mathrm {T}}$	
	190–300$\,\text {GeV}$	$>$500$\,\text {GeV}$
Three-jet mass		
Jet and event selection	0.1	0.1
Jet energy scale	0.3–5.0	0.2–5.5
Jet resolution	0.1–10.0	0.2–6.0
Model dependence in unfolding	0.2–11.0	0.2–5.0
Total systematic uncertainty	0.3–12.7	0.2–7.9
Statistical uncertainty	1.4–14.5	0.7–10.2
Scaled energy of the leading jet		
Jet and event selection	0.1	0.1
Jet energy scale	0.1–1.9	0.1–1.4
Jet resolution	0.2–6.2	0.1–5.4
Model dependence in unfolding	0.1–6.0	0.5–3.6
Total systematic uncertainty	0.8–7.2	1.1–5.6
Statistical uncertainty	1.6–17.2	0.6–14.2
Scaled energy of the second-leading jet		
Jet and event selection	0.1	0.1
Jet energy scale	0.1–2.0	0.1–2.0
Jet resolution	0.1–5.0	0.1–4.2
Model dependence in unfolding	0.4–9.0	0.1–3.5
Total systematic uncertainty	1.0–8.3	0.1–4.6
Statistical uncertainty	1.3–16.4	0.9–8.0
Four-jet mass		
Jet and event selection	0.1	0.1
Jet energy scale	0.4–6.9	0.3–7.0
Jet resolution	0.4–11.7	0.2–4.9
Model dependence in unfolding	0.3–7.0	0.5–8.1
Total systematic uncertainty	0.4–13.7	0.5–11.6
Statistical uncertainty	3.1–30.9	1.4–12.5
Bengtsson–Zerwas angle		
Jet and event selection	0.1	0.1
Jet energy scale	0.1–3.0	0.2–2.4
Jet resolution	0.4–5.4	0.2–5.0
Model dependence in unfolding	0.3–3.5	0.1–6.4
Total systematic uncertainty	1.4–5.9	1.0–8.1
Statistical uncertainty	5.1–8.4	2.8–4.0
Nachtmann–Reiter angle		
Jet and event selection	0.1	0.1
Jet energy scale	0.1–1.0	0.1–1.1
Jet resolution	0.1–4.6	0.2–2.1
Model dependence in unfolding	0.2–2.1	0.4–5.0
Total systematic uncertainty	0.9–5.0	0.9–5.2
Statistical uncertainty	3.4–4.2	1.3–1.6

## Results

### Comparison with models

The normalized differential distributions, corrected for detector effects, are plotted as a function of the three- and four-jet inclusive variables and compared with predictions from the four MC models: pythia 6, pythia 8, MadGraph+pythia6, and herwig++. The variables considered for these comparisons are three-jet mass, scaled energies of the leading and next-to-leading jet in the three-jet sample in the three-jet CM frame, four-jet mass, and the two angles $\chi _{\mathrm {BZ}}$ and $\theta _{\mathrm {NR}}$.

**Fig. 5:**
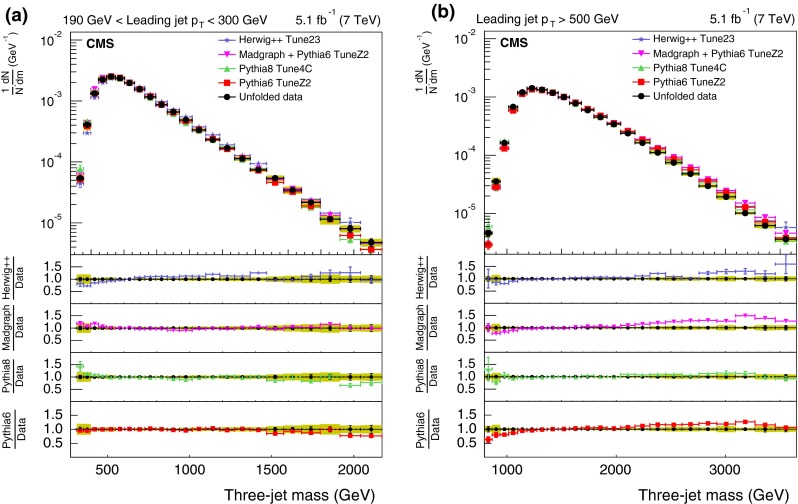
Distribution of the three-jet mass superposed with predictions from four MC models: pythia 6, pythia 8, MadGraph  $+$ pythia 6, herwig++. The distributions are obtained from inclusive three-jet sample with the jets restricted in the $|y |$ region $0.0 <|y |< 2.5$, and with leading-jet $p_{\mathrm {T}}$ between 190 and 300$\,\text {GeV}$ (**a**) or above 500$\,\text {GeV}$ (**b**). The data points are shown with statistical uncertainty only and the bands indicate the statistical and systematic uncertainties combined in quadrature. The *lower panels of each plot* show the ratios of MC predictions to the data. The ratios are shown with statistical uncertainty in the data as well as in the MC, while the *band* shows combined statistical and systematic uncertainties

**Fig. 6:**
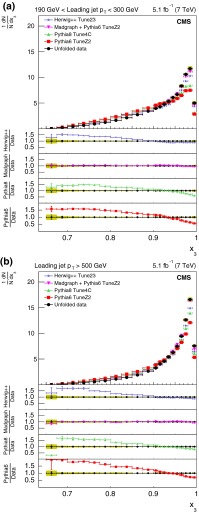
Corrected normalized distribution of scaled energy of the leading-jet in the inclusive three-jet sample. The other explanations are the same as Fig. 5

**Fig. 7:**
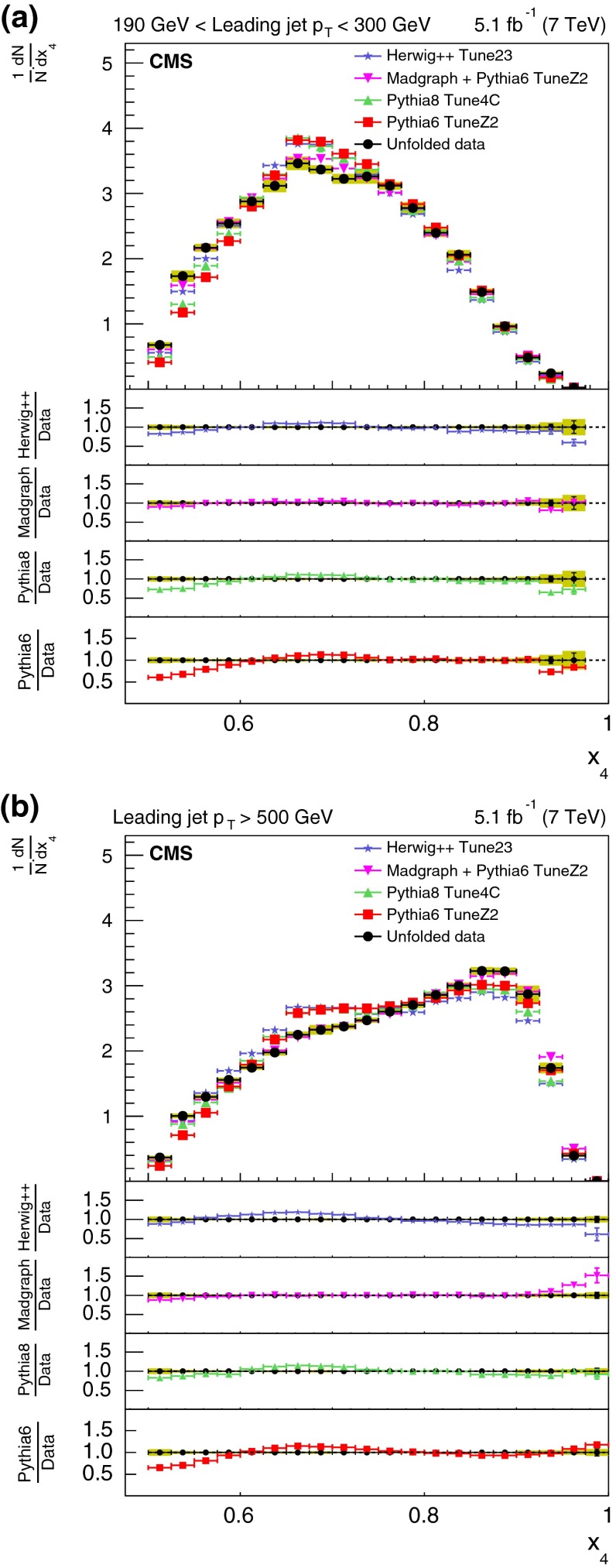
Corrected normalized distribution of scaled energy of the second-leading jet in the inclusive three-jet sample. The other explanations are the same as Fig. 5

**Fig. 8:**
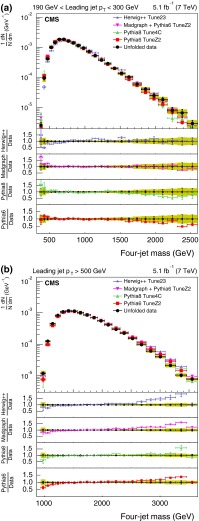
Corrected normalized distribution of four-jet mass. The other explanations are the same as Fig. 5

**Fig. 9:**
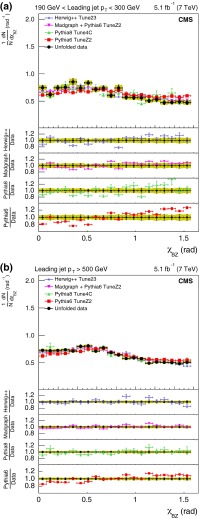
Corrected normalized distribution of the Bengtsson–Zerwas angle. The other explanations are the same as Fig. 5

For the comparison plots (Figs. 5, 6, 7, 8, 9), the upper panel shows the data and the model predictions with the corresponding statistical uncertainty. For the data, the shaded area shows the statistical and systematic uncertainties added in quadrature. The lower panels in each plot show the ratio of MC prediction to the data for each model. Comparisons are made for two different ranges of the leading jet $$p_{\mathrm {T}}$$: $$190 < p_{\mathrm {T}} < 300\,\text {GeV} $$ and $$p_{\mathrm {T}} >500$$$$\,\text {GeV}$$.

Figure 5 shows the normalized corrected differential distribution as a function of the three-jet mass for two ranges of the leading-jet $p_{\mathrm {T}}$. The three-jet mass distribution broadens for larger $p_{\mathrm {T}}$ thresholds. The models show varying degrees of success for the different ranges of leading-jet $p_{\mathrm {T}}$. Most models differ from the data in the low-mass spectrum. The pythia 6 simulation provides a good description of the data in the lower $p_{\mathrm {T}}$ bin, while it has a larger deviation in the higher $p_{\mathrm {T}}$ bin. The mean difference is at the level of 1.8–4.0 %. Predictions from MadGraph  $+$ pythia 6 and pythia 8 agree with the data to within 4.5 %. herwig++ provides the worst agreement among the four models – the mean difference is at the level of 4.0–15 %.

Figure 6 shows the corrected normalized differential distribution as a function of the scaled leading-jet energy in the inclusive three-jet sample. The distributions peak close to 1 and the peaks get sharper for higher leading-jet $p_{\mathrm {T}}$ range. The scaled leading-jet energy $x_3$ is expected to follow a linear rise from $\frac{2}{3}$ to 1 for a phase space model, which has only energy-momentum conservation, while QCD predicts a deviation from linearity at higher values of $x_3$. This feature is observed in the data, particularly for higher $p_{\mathrm {T}}$ bins. Only MadGraph  $+$ pythia 6 provides a consistent description of the data. The agreement improves for the sample with leading-jet $p_{\mathrm {T}}$ above 500$\,\text {GeV}$. The difference between the predictions from MadGraph  $+$ pythia 6 and the data are at the level of 3.5–6.1 %.

Figure 7 shows the corrected normalized differential distribution as a function of the scaled energy of the second-leading jet, $x_4$, in the inclusive three-jet sample. For kinematic reasons, $x_4$ is expected to lie between 1/2 and 1. The distribution peaks around 0.65 for the low $p_{\mathrm {T}}$ threshold sample. The peak shifts to higher values of $x_4$ and the distribution becomes broader for the larger $p_{\mathrm {T}}$ threshold sample. Predictions from MadGraph  $+$ pythia 6 agree with data to within 3.1 %. Predictions from pythia 6 as well as pythia 8 deviate by as much as 10 % or more from the data. Predictions from herwig++ also shows a large deviation at higher $p_{\mathrm {T}}$ bins.

Figure 8 shows comparisons of the corrected normalized differential distribution as a function of the four-jet mass for the four MC models. The distribution broadens at higher minimum $p_{\mathrm {T}}$ value. As can be seen from the figure, herwig++ provides the worst comparison. The average deviations are at the level of 15 % for many of the distributions, particularly for the sample with leading-jet $p_{\mathrm {T}}$ between 190 and 300$\,\text {GeV}$. The level of agreement for the other three MC models is better than 10 % over the entire $p_{\mathrm {T}}$ region.

The sub-leading jets in the four-jet event category are predominantly due to the secondary splitting of partons. In case of gluon splitting, they can be due to a $\mathrm{q} \overline{\mathrm{q}}$ pair or gluons. Both the angular distributions, $\theta _{\mathrm {NR}}$ and $\chi _{\mathrm {BZ}}$, are different for these two scenarios and are representative of the colour factors for these couplings.

Figure 9 shows similar comparisons for the Bengtsson–Zerwas angle. Because the azimuthal angle is not defined for the back-to-back jets, the opening angle between the two leading and two nonleading jets is required to be less than 160$^{\circ }$. As can be seen from the average deviation of the ratios from unity, predictions from MadGraph  $+$ pythia 6 and herwig++ represent the data well, while those from pythia 6 do poorly.

**Fig. 10:**
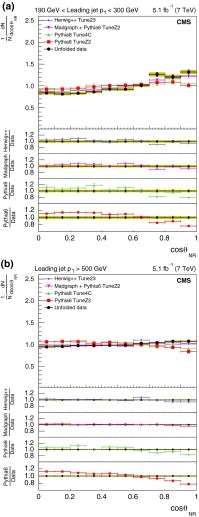
Corrected normalized distribution of the cosine of the Nachtmann–Reiter angle. The other explanations are the same as Fig. 5

Figure 10 shows the corrected normalized differential distribution as a function of the cosine of the Nachtmann–Reiter angle in the inclusive four-jet sample. Most of the models follow the broad features of the data. However, the degree of agreement with data is different among models. MadGraph  $+$ pythia 6 provides the best description of the data; herwig++ with angular ordering in the parton shower is close to the data (the agreement is better than 5 %), while pythia 6 has the largest deviation (the agreement is typically between 10–12 %).

### Effect of hadronization, underlying event, and PDFs

The disagreement between data and the MC models may arise from the implementation of nonperturbative components in the simulation due to the fragmentation model or the choice of PDF set. These effects have been investigated by studying the uncertainties due to hadronization model and PDF parametrization.

The MC models have different ways of modeling the underlying events and hadronization of the partons into hadrons. This may result in different predictions of the distributions of multijet variables depending on whether they are computed at the hadron or at the parton level. This effect has been investigated by studying two different MC models: pythia 6 and herwig++. This is done by evaluating the distributions at the parton and hadron level. pythia 6 uses the Lund string model, while herwig++ uses the cluster model. Also, colour reconnections are done differently in the two models. A generator-level study is carried out for both these models, where the effect of hadronization is studied using distributions from jets at parton- and hadron-level. The ratio of the parton- to the hadron-level distribution is then compared. The mean difference between the two hadronization models is typically less than 5 %.

Comparisons are also made to different tunes of the underlying event models within pythia 6. The tunes (D6T, DW, P0, Z1, Z2, Z2*) [10, 11, 38–40] differ in the cutoff used to regularize the 1/$p_{\mathrm {T}} ^4$ divergence for final-state partons, the ordering of the showers (virtuality ordering vs. $p_{\mathrm {T}}$ ordering), multiparton interaction model, PDFs, and data sets used in the tune. The resulting distributions agree typically within 5 %, so the disagreements with the data cannot be fully explained by this effect.

The MC models use CTEQ6 as the default PDF parametrization. There are many different PDF sets, which are based on different input data, assumptions, and parametrizations. Thus any calculation of a cross section or distributions in the simulation depends on the choice of PDF set. Also, each PDF set has its own errors from its parametric assumptions and data input to fitting. The effect of the PDF set choice on the multijet variables is calculated according to the recommendation of PDF4LHC group [41, 42]. Since comparisons are made only with leading order Monte Carlo models in this paper, only two leading order PDF sets are used in this comparison: CTEQ6l and MSTW2008lo68cl [43]. The uncertainties are found to be typically at the level of 1.0–2.0 % depending on the variable type and $p_{\mathrm {T}}$ range considered.

## Summary

Distributions of topological variables for inclusive three- and four-jet events in pp collisions measured with the CMS detector at a centre-of-mass energy of 7$\,\text {TeV}$ were presented using a data sample corresponding to an integrated luminosity of 5.1$\,\text {fb}^{-1}$. The distributions were corrected for detector effects, and systematic uncertainties were estimated. These corrected distributions were compared with the predictions from four LO MC models: pythia 6, pythia 8, herwig++, and MadGraph  $+$ pythia 6.

Distributions of three- and four-jet invariant mass from all models show significant deviation from the data at high mass. The fact that all models have a common PDF suggests that the PDF errors at high mass are underestimated. The PDFs at high invariant mass have recently been constrained by CMS using dijet $p_{\mathrm {T}}$ distributions[44].

The MadGraph simulations are based on tree-level calculations for two-, three-, and four-parton final states, while pythia and herwig++ can have only two partons in the final state before showering. Not surprisingly, the three-jet predictions of MadGraph  $+$ pythia 6 give a more consistent description of the distributions studied in this analysis. The notable exception is at high $x_4$ (the next-to-leading jet), where two jets carry most of the CM energy. The difference is probably due to a double counting of three-parton with two-parton (with a parton from showering) final states.

The pythia and herwig++ models give poor descriptions of the energy fractions in the three-jet final state. In particular, the distributions of $x_3$ (the leading jet) show large shape differences between data and theory that are inconsistent with PDFs or hadronization model uncertainties. Since the distributions from MadGraph  $+$ pythia 6 agree with those from the data, the discrepancies with pythia and herwig++ are likely due to missing higher multiplicity ME, which are present in MadGraph.

All the models compared in this study do remarkably well describing the four-jet Bengtsson–Zerwas angle. The pythia models have some systematic deviation from the data in describing the Nachtmann–Reiter angle. Parton showers with angular ordering, as implemented in herwig++, yield a better agreement with the measured data for these angular variables.
